# Two Centrins and Their Posttranslational Modification Modulate the Cell Cycle of *Giardia lamblia*

**DOI:** 10.1002/mbo3.70038

**Published:** 2025-07-29

**Authors:** Hye Rim Yeo, Mee Young Shin, Juri Kim, Soon‐Jung Park

**Affiliations:** ^1^ Department of Tropical Medicine, Institute of Tropical Medicine Yonsei University College of Medicine Seodaemun‐gu Seoul South Korea

**Keywords:** cell cycle, centrin, *Giardia lamblia*, SUMOylation

## Abstract

Centrins, Ca^2+^‐binding proteins conserved in eukaryotes, are the key components of the microtubule‐organizing center. *Giardia lamblia* possesses two centrins (GL50803_6744: centrin 1; GL50803_104685: centrin 2) localized in the basal bodies during cell division. *G. lamblia* centrin 2 (Glcent2) is also found in the nuclei of interphase *Giardia*, with its N‐terminal half being necessary for this localization. Morpholino‐mediated knockdown of Glcents resulted in abnormal nuclear positioning and cytokinesis, causing cell malformations, including ventral discs and flagella defects. Small ubiquitin‐like modifier (SUMO)ylation is a posttranslational modification, which modulates several cellular processes. Here, we demonstrated that Glcents are substrates of SUMO through in vitro SUMOylation and immunoprecipitation experiments. Additionally, treatment of *Giardia* with ginkgolic acid, which inhibits the E1 enzyme of the SUMO pathway, and CRISPRi‐mediated inhibition of *G. lamblia* Ubc9, the E2 conjugation enzyme involved in SUMOylation, resulted in defects in the localization of Glcents. Blocking SUMOylation resulted in the arrest of *Giardia* cells and conformational changes, including alterations in the ventral disc shape, posterolateral flanges, and peripheral vesicles. Taken together, we demonstrated that Glcents function in *Giardia* cell cycle progression and morphogenesis, with the activity of both Glcents being modulated by SUMOylation.

## Graphical abstract

Two *Giardia lamblia* centrins (Glcents) play a role in cell cycle progression and morphogenesis of *Giardia*. Both Glcents are substrates of GlSUMO, suggesting a role of SUMOylation in their proper localization. SUMOylation is also involved in regulating vacuole formation in *Giardia*.

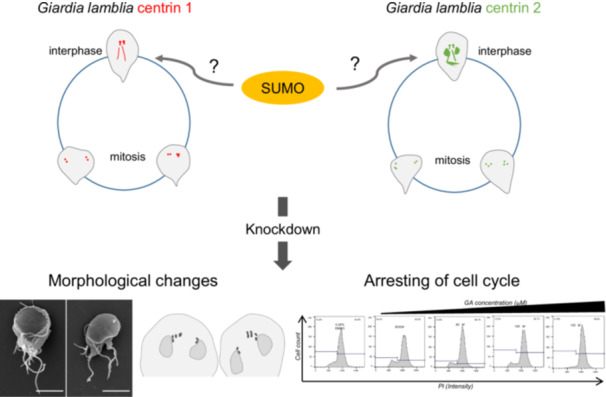

## 1 Introduction

Centrins are a group of proteins conserved in eukaryotic cells, which have four helix‐loop‐helix domains for Ca^2+^‐binding (Friedberg 2006; Pedretti et al. 2021). They have been identified in structures connected with microtubule‐organizing centers (MTOCs), that is, the spindle pole body (SPB) of budding yeasts, basal bodies of cells with cilia or flagella, and the centrosomes of nonciliated cells (Spang et al. 1993; Satisbury 1995; Kilmartin 2003). MTOC dysfunction is linked to various human diseases, including blindness, male infertility, cancer, and obesity (Wingfield and Lechtreck 2018).

The major role of centrins is in the duplication of MTOCs (Pedretti et al. 2021). Temperature‐sensitive and conditional mutations in the budding yeast centrin Cdc31p demonstrated defects in SPB duplication and G2/M arrest (Baum et al. 1986; Ivanovska and Rose 2001). RNA interference (RNAi)‐mediated knockdown or mutation of the *Chlamydomonas* centrin Vlf2 reduced the number of basal bodies (Taillon et al. 1992; Koblenz et al. 2003). When modified, centrins are known to function in other cellular processes. *Homo sapiens* centrin 2 (Hscent2) is translocated to the nucleus (Middendorp et al. 2000), where it functions in nucleotide excision repair (NER). Hscent2 is modified by small ubiquitin‐like modifier (SUMO)ylation, which is critical for its function in NER (Klein and Nigg 2009).

SUMOylation is a kind of posttranslational modification found in eukaryotes (Geiss‐Friedlander and Melchior 2007). Three enzymes are involved in the transfer of SUMO onto its substrates (Hochstrasser 2000). In an ATP‐dependent manner, the GG motif in the C‐terminal portion of SUMO forms a thioester bond with the cysteine in the Uba2 subunit of the heterodimer E1 enzyme (Aso1/Uba2) (Wang and Dasso 2009; Johnson 1997). Ubc9, the E2 conjugating enzyme, transfers SUMO from Uba2 onto itself or substrates (Desterro et al. 1997; Lee et al. 1998). The E3 ligase facilitates the conjugation of SUMO onto substrates (Geiss‐Friedlander and Melchior 2007). SUMOylated proteins differentially participate in diverse cellular processes, which include cell cycle control, DNA repair, transcriptional regulation, and nucleocytoplasmic transport of specific proteins (Wasik and Filipek 2014; Vertegaal 2022).

*Giardia lamblia* is a parasitic protozoan that causes giardiasis, leading to diarrhea and nutrient malabsorption in infected hosts. Its life cycle consists of two stages, the trophozoite and cyst, which represent the multiplied and infectious forms, respectively (Adam 2021). *G. lamblia* centrin1 (Glcent1: GL50803_6744) and Glcent2 (GL50803_104685) were previously described by Lauwaet et al. (2011). These proteins were found to be expressed in the basal bodies between the two nuclei of *Giardia* trophozoites through immunofluorescence assays (Meng et al. 1996; Corrêa et al. 2004; Benchimol 2005; Sagolla et al. 2006) and by *Giardia* expressing epitope‐tagged Glcentrins (Lauwaet et al. 2011).

A single *sumo* gene (GL50803_7760) and sets of genes encoding SUMO‐activating (GL50803_10661 and 6288), SUMO‐conjugating (GL50803_24068), a SUMO protease (GL50803_16438), and two putative SUMO ligases (GL50803_11930 and 10261) were identified in *G. lamblia* database (https://giardiadb.org/giardiadb/app; Vranych et al. 2012; Castellanos et al. 2020). Depletion of *G. lamblia* SUMO (GlSUMO) using RNAi resulted in cell shape and adhesive disc alterations (Di Genova et al. 2016). The currently known substrates of *Giardia* SUMO are *α*‐tubulin (Di Genova et al. 2016) and arginine deiminase, a metabolic enzyme that changes arginine to citrulline (Touz et al. 2008; Vranych et al. 2014).

This study focused on the roles of the two Glcents in the cell cycle control and morphogenesis of *Giardia* cells. We attempted to differentiate these two Glcents in terms of their localization and function. Based on SUMOylation of Hscent2 (Klein and Nigg 2009), we also investigated whether Glcents are modified by SUMO.

## 2 Materials and Methods

### 2.1 *Giardia lamblia* Cultivation Conditions

A modified TYI‐S‐33 medium (1% yeast extract, 2% casein digest, 0.2% cysteine, 1% glucose, 0.02% ascorbic acid, 0.06% KH_2_PO_4_, 0.2% K_2_HPO_4_, 0.2% NaCl, pH 7.1), supplemented with 10% heat‐inactivated calf serum and 0.75 mg/mL bovine bile (Gibco) (Keister 1983) was used to culture *Giardia* trophozoites (ATCC 30957, Strain WB, American Type Culture Collection) at 37°C. After transfection, the resulting strains were enriched and grown in TYI‐S‐33 medium with 50 μg/mL puromycin or 600 μg/mL G418.

### 2.2 Formation of a *Giardia* Strain Making Epitope‐Tagged *G. lamblia* Centrin (Glcent) Proteins

Information on the primers and plasmids included in this study is listed in Tables 1 and 2, respectively. A 483‐bp DNA fragment of the *glcent1* gene with a 200‐bp its own upstream promoter region was amplified from *Giardia* genome by PCR using the primers 6744‐P‐F and 6744‐R. NotI and XhoI restriction sites were utilized to integrate the *glcent1* DNA into pKS‐3HA.NEO (Gourguechon and Cande 2011) to generate pGlcent1‐HA.NEO. A PCR product with the promoter region and an open reading frame (ORF) of *glcent2* was generated with 104685‐P‐F and 104685‐R primers, after which it was inserted into the XhoI and NotI enzyme sites of pKS‐3HA.NEO to produce pGlcent2‐HA.NEO. In addition, the *glcent2* DNA obtained from pGlcent2‐HA.NEO was inserted into the NotI and AflII restriction sites of pKS‐3myc.PAC (Kim et al. 2017) to produce pGlcent2‐myc.PAC.

To make the N‐terminal truncated Glcent2 protein, a 312‐bp DNA fragment encoding the 1–104 amino acid (aa) region of Glcent2 and a 200‐bp promoter was amplified from pGlcent2‐HA.NEO using the primers 104685‐P‐F and 104685‐N‐4H‐R, and the resulting PCR product was inserted into pKS‐3HA.NEO to produce pGlcent2‐N‐4H. To make the C‐terminus of Glcent2 with its own promoter, two DNA fragments were prepared: a vector containing the *glcent2* promoter fragment was amplified from pGlcent2‐HA.NEO by PCR using p104685‐HA‐F and p104685‐HA‐R, and an insert fragment, the C‐terminal 238‐bp of *glcent2*, which was amplified from pGlcent2‐HA.NEO by PCR using 104685‐C‐4H‐F and 104685‐C‐4H‐R. The two fragments were then ligated using the in‐fusion method to obtain the pGlcent2‐C‐4H plasmid.

A shuttle plasmid containing the myc‐tag and a neomycin resistance (*neo*) cassette was constructed. The vector, excluding the puromycin resistance cassette, was synthesized on pKS‐3myc.PAC by PCR with PAC‐vector‐F and PAC‐vector‐R primers. The *neo* cassette was obtained from pKS‐3HA.NEO by PCR using the NEO‐F and NEO‐R primers. They were ligated using the in‐fusion method, resulting in plasmid pKS‐3myc.PAC. A DNA fragment containing 576‐bp of the *glubc9* gene (GL50803_24068) and 150‐bp of its own promoter was amplified from the *Giardia* genome with the primers 24068‐P‐F and 24068‐R and added into pKS‐3myc.NEO to produce pGlubc9‐myc.NEO. The DNA sequences of these plasmids were confirmed by Macrogen (Seoul, Korea).

Transfection of these plasmids into *Giardia* cells was conducted by electroporation at 700 Ω, 350 V, and 1000 μF (BioRad). The resulting transgenic trophozoites were primarily enriched in a medium supplemented with 150 μg/mL G‐418 and then followed by incubation with 600 μg/mL G418. Presence of HA‐tagged Glcents was monitored by western blot analysis, for which *Giardia* with pKS‐3HA.NEO served as the control.

### 2.3 Western Blotting

Various *G. lamblia* cells (those harboring pKS‐3HA.NEO, pGlcent1‐HA.NEO, or pGlcent2‐HA.NEO) were sonicated in a buffer (1% NP‐40, 150 mM NaCl, 50 mM Tris–HCl, and 10 mM N‐ethylmaleimide, pH 7.4) with protease inhibitors supplied by GenDEPOT. Sodium dodecyl sulfate‐polyacrylamide gel electrophoresis (SDS‐PAGE) was applied to analyze 10–20 μgs of these cell extracts, which were transferred onto a polyvinylidene fluoride filter (Millipore). The filter was then reacted with anti‐HA (1:1000 dilution, H3663, Sigma‐Aldrich) or anti‐myc antibodies (1:1000 dilution, Thermo Scientific) in a blocking solution [5% skim milk, Tris‐buffered saline (TBS; 150 mM NaCl, and 50 mM Tris‐Cl, pH 7.5), and 0.05% Tween 20]. After a subsequent reaction with horseradish peroxidase (HRP)‐combined secondary antibodies, a chemiluminescence system (GE HealthCare) was employed to detect immunoreactive proteins. The filter was incubated in a buffer for stripping (Thermo Scientific) and reacted with polyclonal antibodies (1:10,000 dilution), which were raised against protein disulfide isomerase 1 (GL50803_29487) of *G. lamblia* (GlPDI1) (Kim et al. 2017).

### 2.4 Immunofluorescence Assay

Various *Giardia* trophozoites (cells producing HA‐tagged Glcents, cells treated with SUMOylation inhibitor, and Glubc9 knockdown cells) were kept on ice for 15 min, followed by harvest at 4°C for 15 min. The cell pellets were rinsed three times with phosphate‐buffered saline (PBS) buffer, which was composed of 2.7 mM KCl, 137 mM NaCl, 1.8 mM KH_2_PO_4_, and 10 mM Na_2_HPO_4_ at pH 7.4. The cells were then fixed with PBS containing 100 mM 3‐maleimidobenzoic acid N‐hydroxysuccinimide ester, 100 mM ethylene glycol‐bis‐succinimidyl succinate, and 2% paraformaldehyde for 30 min at room temperature (RT). The fixed cells were adhered to the glass slides coated with poly‐l‐lysine for 10 min, and then followed by 10 min‐incubation at RT in the presence of 0.5% Triton X‐100. The slides were reacted in PBS/3% BSA for 30 min, undergoing a series of incubations with primary and secondary antibodies. ProLong antifade (Invitrogen) containing 4, 6‐diamino‐2‐phenylindole was added to the cells for observation under an LSM980 Zeiss confocal microscope.

The antibodies used for this study are listed as below, together with the information on immunized hosts, vendors, references, and dilution‐folds: (1) rat antibodies against HA epitope (1:100, clone 3F10, Roche), Glγ‐tubulin (1:100, Kim and Park 2019a), anti‐GlSUMO (1:100), Glγ‐giardin (1:200, Kim and Park 2019b), and Glcent2 (1:400, Kim and Park 2019b), (2) mouse antibodies against myc epitope (1:100, Thermo Scientific), acetylated‐α‐tubulin (1:800, T7451, clone 6‐11B‐1, Sigma‐Aldrich), HA epitope (1:100, H3663, Sigma‐Aldrich), (3) Alexa Fluor 555‐ or Alexa Fluor 488‐conjugated donkey antibodies against anti‐rat IgG, or anti‐mouse IgG, respectively (1:100, Molecular Probes).

The mean fluorescence intensity (MFI) of the basal bodies or nuclei was measured using ImageJ (ver. 1.53e, http://imgej.nih.gov/ij). MFI values were analyzed, and the results are presented as bar graphs using SigmaPlot (SigmaPlot ver. 9.0; Systat Software Inc.).

### 2.5 Knockdown Experiment Using Morpholinos

Expression of Glcent1 or Glcent2 was inhibited using a morpholino based on a previously described method (Kim et al. 2023). Specific morpholinos for Glcent1 and Glcent2 were synthesized using GeneTools, and Table 1 presents the information on their nucleotide sequences. A control morpholino was also made to have nonspecific sequences. Each morpholino at 100 μM was electroporated into *Giardia* trophozoites (5 × 10^6^ cells). The resulting transfectants cultivated for varying periods from 6 to 24 h were analyzed by western blot with antibodies against the HA‐epitope to monitor the amount of Glcents. At 24 h posttransfection, these cells were reacted with Giemsa, whereafter their nuclear phenotypes and the percentage of splitting cells were measured. The cells transfected with control or anti‐*glcents* morpholinos were collected after 24 h, and their morphology was examined using differential interference contrast imaging, scanning electron microscopy, and transmission electron microscopy. At least three transfections were conducted, and the displayed figures are chosen among them.

**TABLE 1:** Primers used in this study.

Primers	Sequences (5′ to 3′)
For epitope‐tagged constructs	
6744‐P‐F	ATAAGAATGCGGCCGCCTGTCTGCGATCTTCCAGGGTGC (*NotI*)
6744‐R	CGCCTCGAGTAGAGGGACGTGCGGCGCATTATCC (*XhoI*)
104685‐P‐F	ATAAGAATGCGGCCGCACTTTATCGTGTAACCGGCGGGCCCA (*NotI*)
104685‐R	CGCCTCGAGGAGAAAGCACTTGTGGACCTTAAA (*Xhol*)
104685‐N‐4H‐R	CGCCTCGAGTCACGGTTACTTATCTTCTCTATC (*XhoI*)
P104685‐HA‐F	tcgagcttaaggaatatccttatgacgt
P104685‐HA‐R	TTAAATTTAGGATCTGAAGTTTGAGAAGGTTTTATGT
104685‐C‐4H‐F	ACTTCAGATCCTAAATTTAAAGTAACCGTGACCCCACAG
104685‐C‐4H‐R	aggatattccttaagctcgaAGAGAAAGCACTTGTGGACC
PAC‐vector‐F	gggctgaagtgaatatttaccttttccg
PAC‐vector‐R	gattttcttgttttagggttagttttttagaaaaggcg
NEO‐F	taaaacaagaaaatcatgattgaacaagatggattgc
NEO‐R	tattcacttcagccctcagaagaactcgtcaagaaggc
24068‐P‐F	ATAAGAATGCGGCCGCATCTCTTTGGGGGTGGCCTTATC (*NotI*)
24068‐R	AGCCTCTCGAGATCTATGGCTATGTCGTCATTC (*XhoI*)
For morpholino oligo sequences	
Anti‐*glcent1*	TGCCATAGGAGATTTTGGACTGCCA
Anti‐*glcent2*	GCGCCTATGGCCGCTCTATTCATTT
Control	CCTCTTACCTCAGTTACAATTTATA
For recombinant protein constructs	
6744‐His‐F	GCCGAATTCGGAACCCAAGCGGGAGGAGTTGAAG (*EcoRI*)
6744‐His‐R	ATAAGAATGCGGCCGCATAGAGGGACGTGCGGCGCATTATC (*NotI*)
7760‐F	CCGGAATTCATGGATGACGAAGGAGACGT (*EcoRI*)
7760‐gg‐R	CGCCTCGAGCTAGCCGCCAATCTGATTTCGCAT (*XhoI*)
For *ubc9* knockdown guide RNA	
Control‐gRNA‐F	caaaCCTCCTTACCTCAGTTAT
Control‐gRNA‐R	aaacATAACTGAGGTAAGGAGG
Ubc9‐gRNA29‐F	caaaAAGAAAGAAAACACATAAAA
Ubc9‐gRNA29‐R	aaacTTTTATGTGTTTTCTTTCTT
Ubc9‐gRNA43‐F	caaaATAAAAAGGGAGCTTCCGCC
Ubc9‐gRNA43‐R	aaacGGCGGAAGCTCCCTTTTTAT
For confirming mRNA expression	
actin‐RT‐F	GTCCGTCATACCATCTGTTC
actin‐RT‐R	GTTTCCTCCATACCACACG
Ubc9‐RT‐F	CCATTGTTGAAGAAAATCCC
Ubc9‐RT‐R	TCAATCTATGGCTATGTCGTCAT

*Note:* Underlined bases indicate a restriction enzyme site.

### 2.6 Giemsa Staining

To examine the nuclear phenotypes, *Giardia* trophozoites with Glcents or Glubc9 knockdown were stained using Giemsa stain. Fixation of the cells was performed by a 10‐min incubation with 100% methanol at −20°C, as previously described (Park et al. 2021). After staining with 10% Giemsa, the cells were rinsed and analyzed under an Axiovert 200 microscope (Zeiss). More than five hundred cells were observed to assess their nuclear position and number.

### 2.7 Scanning Electron Microscopy (SEM)

Morpholino‐treated, inhibitor‐treated, and CRISPRi‐mediated knockdown cells were reacted in a fixative buffer (2% paraformaldehyde, 2% glutaraldehyde, 0.5% CaCl_2_/0.1 M cacodylate, pH 7.4), rinsed in 0.1 M cacodylate, and then incubated in the same buffer with 1.33% osmium tetroxide. After that, water was removed from the cells with an ascending, gradual series of ethanol (50%–100%) in an EM CPD300 dryer (Leica). Rehydration with isoamyl alcohol and the subsequent coating with gold of 300 Å in an EM ACE600 ion coater (Leica) were conducted on the prepared samples prior to being examined with a field‐emission SEM (Zeiss).

### 2.8 Transmission Electron Microscopy (TEM)

Morpholino‐treated, inhibitor‐treated, and CRISPRi‐mediated knockdown cells were reacted with 2% paraformaldehyde, 2% glutaraldehyde, and 0.1 M phosphate, pH 7.4. Thereafter, these cells were incubated with 1% OsO_4_ for 2 h and dried with a gradually increasing amount of ethanol (50%–100%), followed by infiltration with propylene oxide. After being embedded using a kit (Poly/Bed 812, Polysciences), a 12 h‐polymerization of the samples was carried out in a TD‐700 oven (Dosaka EM) at 65°C. Thin 80‐nm slices of these cells were contrast‐stained with lead citrate and 6% uranyl acetate (Thermo Scientific). The samples were sliced further using an EM UC‐7 (Leica) and conveyed onto copper grids. They were examined under a TEM (JEM‐1011, JEOL) at 80 kV.

For longitudinally sectioned TEM, cells were collected and deposited onto coverslips in a 6‐well culture plate and incubated at 37°C for 1 h inside a GasPak EZ bag (BD Biosciences). After polymerizing the specimen, the coverslips were dissolved from the epon layer by immersion in 35% hydrofluoric acid (Sigma‐Aldrich).

### 2.9 Construction of Histidine‐Tagged Glcent1 and GLSUMO Protein

To make recombinant Glcent1 (rGlcent1), a 486‐bp *glcent1* DNA was synthesized from the *Giardia* genome with 6744‐His‐F and 6744‐His‐R (Table 1). The resulting DNA fragment was added to pET21b (Novagen) to generate p6744‐HIS. A 306‐bp *glsumo* DNA was made using 7760‐F and 7760‐gg‐R, then added into pET28a (Novagen). The resulting construct, pHIS‐SUMOgg, was verified through DNA sequencing performed by a sequencing service (Macrogen).

### 2.10 Preparation of Recombinant Protein

The plasmids for recombinant proteins were maintained in *Escherichia coli* BL21 (DE3). In order to induce the expression of the recombinant proteins, 0.5 mM isopropyl‐β‐d‐thioglactopyranoside (IPTG, Sigma‐Aldrich) was added to the resulting cells cultivated for 3 h at 20°C. After being harvested and resuspended in a buffer (50 mM NaH_2_PO_4_, 300 mM NaCl, pH 7.0), the cells were broken down via sonication in the presence of 1% Triton X‐100. The soluble proteins were prepared by a 20‐min centrifugation at 14,000 *g*, and then bound with an affinity resin (TALON, Clontech). After a series of washes with the buffer mentioned above, the recombinant proteins were eluted with 150 mM imidazole, which was removed from the protein by dialysis in PBS.

### 2.11 Formation of Polyclonal Antibodies Specific to Recombinant GlSUMO Protein

One millimolar IPTG was used to induce the expression of the GlSUMO protein tagged with histidines in *E. coli*. The resulting recombinant protein was administered to Sprague–Dawley rats (female, 2‐week‐old) to make antibodies (Kim and Park 2019b), which were purified via an affinity chromatography using the protein A/G resin. All experiments using animals were permitted by institutional directions and legal requisitions (approval number, 2012‐0264‐1; IACUC 2017‐0015).

### 2.12 In Vitro SUMOylation

The SUMOylation assays of rGlcent proteins were conducted using a BML‐UW8955 kit (Enzo), as directed by the manufacturer. Briefly, the human SUMO‐activating enzyme E1, human conjugation enzyme E2, *Homo sapiens* SUMO‐1 (HsSUMO‐1), and 5 μg rGlcents were mixed in a volume of 20 μL with or without ATP. The assays were maintained for 1 h at 37°C and then stopped with the addition of an equal volume of 2× loading buffer (used for denaturing gels). The reactants were separated on a 12% denaturing gels and transferred onto a PVDF filter (Millipore). Another set of assays was performed using 2.5 μg purified rGlSUMO protein instead of HsSUMO‐1. The filter was reacted with mouse monoclonal antibodies detecting histidine residues (1:10,000 dilution, IG Therapy), rabbit polyclonal anti‐HsSUMO‐1 antibodies (1:1000 dilution, BML‐PW8330‐0025, Enzo), or polyclonal rat anti‐GlSUMO antibodies (1:1000). After the reaction with HRP‐combined secondary antibodies, a chemiluminescence kit supplied by GE HealthCare was used to detect the immunoreactive proteins.

### 2.13 Treatment With a SUMOylation Inhibitor, and Viability Assay

To determine the 50% inhibitory concentration (IC_50_) of the SUMOylation inhibitor, namely ginkgolic acid (GA, S9432, Selleckchem), the *Giardia* cells (5 × 10^5^ cells mL^−1^) were exposed to various concentrations (0–100 μM) of GA, and then incubated in a culture plate of 96‐well at 37°C, and 5% CO_2_ for 24 h, and cell viability was determined using a kit (ab129732, Abcam) as instructed by the manufacturer. Briefly, only the attached cells were recovered and rinsed with PBS. Resazurin (10%) was added to the well and left to react for 6 h, and the relative fluorescent units were then measured at wavelengths of 544 (for excitation) and 599 nm (for emission) using a microplate reader (Molecular Devices). The IC_50_ was defined via nonlinear regression based on a sigmoid curve (SigmaPlot 9.0). Each experiment was repeated three times.

A group of *Giardia* trophozoites was reacted with different amounts of GA (60, 80, 100, and 120 μM), and their DNA ploidy was determined by flow cytometry. As a standard, the cells were exposed to 0.24% dimethyl sulfoxide (DMSO) for 24 h, and then processed in the same manner. In the subsequent experiment, *Giardia* was exposed to 120 μM GA for various times (from 3 h to 36 h), and the cells treated with 0.24% DMSO for the same time periods (control cells) were investigated by flow cytometry.

### 2.14 Flow Cytometry

Evaluation of DNA ploidy by flow cytometry was conducted on GA‐ as well as DMSO‐exposed *Giardia*, as described (Kim et al. 2014). Three volumes of cell fixative (20 mM Na_2_HPO_4_, 200 mM sucrose, 1% Triton X‐100, and 40 mM citric acid, pH 3.0) were added to the cells resuspended in the culture medium and kept for 5 min at RT, and the samples were diluted with PBS with 125 mM MgCl_2_. Prior to flow cytometric analysis, the samples were subjected to 30‐min incubation with 2.5 μg RNase A (Sigma‐Aldrich) and 10 μg/mL propidium iodide at 37°C. The FlowJo software provided by FlowJo LLC was applied to analyze the data. The histograms displayed in the results were derived from three separate experiments.

### 2.15 CRISPRi‐Mediated Knockdown

A plasmid encoding an endonuclease‐deficient Cas9 (dCas9) and guide RNA (gRNA)‐expression cassette was synthesized corresponding to a previous publication (McInally et al. 2019) (Macrogen), and the resulting plasmid was named pdCas9.PAC (Table 2). Specific gRNAs for the *glubc9* gene were synthesized using tools available at http://www.rgenome.net/cas-designer/. They were devised in relation to the NGG PAM sequences, which were suggested by the *G. lamblia* database (GCA_000002435.1). The list of oligonucleotides encoding *glubc9* and the control gRNA is presented in Table 1. Using these oligonucleotides, double‐stranded gRNAs were made and added into the BbsI of pdCas9.PAC. The gRNAs for *glubc9* were designed to target the nucleotides corresponding to +29 and +43 of the Glubc9 coding region. The resulting gRNA‐expressing plasmids were pdCas9‐ubc9‐g29 and pdCas9‐ubc9‐g43. In order to evaluate the effects of Glubc9 knockdown, a plasmid pdCas9‐gCont making the control gRNA was constructed and included in the experiments (Table 2). These constructs were electroporated into *Giardia* cells harboring the expression plasmid for myc‐tagged Glubc9, and the transfectants were selected by growing them in culture medium containing puromycin.

**TABLE 2:** Strains and plasmids used in this study.

Strain	Relevant characteristics	Source or References
*E. coli*		
DH5α	*supE44 ΔlacU169* (Φ80 *lacZ ΔM15*) *hsdR17 recA1 endA1 gyrA96 thi‐1 relA1*	Invitrogen
BL21 (DE3)	*F′, ompT, hsdSB(rB‐mB‐) gal, dcm (DE3)*	Invitrogen
Plasmids		
pKS‐3HA.NEO	Shuttle vector, Amp^R^, *neo* gene	Gourguechon and Cande (2011)
pGlcent1‐HA.NEO	pKS‐3HA.NEO, 200‐bp own promoter region and 483‐bp encoding *Giardia centrin1* gene (GiardiaDB: GL50803_6744)	This study
pGlcent2‐HA.NEO	pKS‐3HA.NEO, 200‐bp own promoter region and 528‐bp encoding *Giardia centrin2* gene (GiardiaDB: GL50803_104685)	This study
pGlcent2‐N‐4H	pKS‐3HA.NEO, 200‐bp own promoter region and 312‐bp encoding N‐terminus of *Giardia centrin2* gene (GiardiaDB: GL50803_104685)	This study
pGlcent2‐C‐4H	pKS‐3HA.NEO, 200‐bp own promoter region and 238‐bp encoding C‐terminus of *Giardia centrin2* gene (GiardiaDB: GL50803_104685)	This study
pKS‐3myc.PAC	Shuttle vector, Amp^R^, *puromycin* gene	Gourguechon and Cande (2011)
pGlcent2‐myc.PAC	pKS‐3myc.PAC, 200‐bp own promoter region and 528‐bp encoding *Giardia centrin2* gene (GiardiaDB: GL50803_104685)	This study
pET21b	Expression vector for a histidine‐tagged protein, Amp^R^	Novagen
p6744‐HIS	pET21b, 486‐bp encoding *glcentrin1* (GiardiaDB: GL50803_6744)	This study
pET28b	Expression vector for a histidine‐tagged protein, K an^R^	Novagen
pETcentrin	pET28b, 531‐bp encoding *glcentrin2* (GiardiaDB: GL508003_104685)	Kim and Park (2019b)
pHIS‐SUMOgg	pET28b, 306‐bp encoding *glf. SUMO* (GiardiaDB: GL508003_7760)	This study
pKS‐3myc.NEO	Shuttle vector, Amp^R^, *neomycin* gene	This study
pGlubc9‐myc.NEO	pKS‐3HA.NEO, 150‐bp own promoter region and 576‐bp encoding *Giardia ubc9* gene (GiardiaDB: GL50803_24068)	This study
pdCas9.PAC	Amp^R^, Puromycin resistance cassette, HA‐tagged *dCas9* gene from *Giardia* maltose dehydrogenase promoter, guide RNA expressing cassette	This study
psCas9‐gCont	pdCas9.PAC, control guide RNA expressing cassette	This study
pdCas9‐ubc9‐g29	pdCas9.PAC, Ubc9 guide RNA expressing cassette	This study
pdCas9‐ubc9‐g43	pdCas9.PAC, Ubc9 guide RNA expressing cassette	This study

Abbreviations: Amp, ampicillin; Kan, kanamycin; ^R^, resistant; HA, hemagglutinin.

### 2.16 Determination of Transcript Levels via Quantitative Real‐Time PCR (qRT‐PCR)

Total RNAs were extracted from *Giardia* expressing dCas9 and control or glubc9 gRNA using TRIzol (Thermo Scientific) following the provided protocols. The Improm‐II Reverse Transcription System of Promega was applied to five micrograms of RNA to produce complementary DNA (cDNA). Reaction of these cDNAs with the LightCycler 480 SYBR Green I Master Kit (Roche) was conducted in the following conditions: an initial 5‐min reaction at 95°C, followed by 45 cycles comprised with a 30‐s step at 94°C, a 30‐s step at 56°C, and a 30‐s step at 72°C. Information related to the primers for qRT‐PCR is presented in Table 1. The transcript levels of actin‐related gene (GL50803_15113) in *G. lamblia* were used for normalization (Kim and Park 2019b). Software from Roche (version LSC480 1.5.0.39) was applied to these data to define the crossing‐point values, which are efficient for relative quantification among the samples.

### 2.17 Statistical Analyses

Data are presented as the means of at least three separate experiments. Especially, pairwise comparisons for statistical significance were conducted based on the Student's *t*‐test (SigmaPlot 9.0). Differences with *p*‐values < 0.05 were considered significant. Comparisons with two asterisks in the figures denote *p*‐values < 0.01, and a single asterisk demonstrates 0.01 < *p*‐values < 0.05.

## 3 Results

### 3.1 Two Glcentrins, Glcent1 and Glcent2, Co‐Localize at Basal Bodies

The expression of Glcent1‐HA (GL50803_6744) was observed in the extracts of transgenic *Giardia* carrying pGlcent1‐HA.NEO via western blot using antibodies specific to the HA epitope (Figure 1a). *Giardia* cells expressing Glcent1‐HA were examined via immunofluorescence assays (IFAs) using anti‐HA to detect Glcent1. In addition, these cells were stained with antibodies against anti‐acetylated α‐tubulin for mitotic spindles or Glγ‐tubulin for basal bodies, which serve as markers of dividing cells. In interphase cells, red fluorescence was mainly observed as basal bodies between the nuclei and the cytoplasmic axonemes of the posterolateral flagella. At various stages of mitosis, Glcent1 was present in basal bodies connected by mitotic spindles, which were labeled with green fluorescence in IFAs using antibodies against acetylated α‐tubulin (Figure 1b). Additionally, the basal bodies in dividing cells were visualized using antibodies against Glγ‐tubulin, which was found in basal bodies of *Giardia* cells (Kim and Park 2019a; Figure 1c). The mean fluorescence intensity (MFI) of Glcent1 in basal bodies was 183 (*n* = 34), and in the nuclei, it was 40 (*n* = 39).

FIGURE 1Expression and localization of *Giardia lamblia* centrins (Glcents) in *Giardia* expressing epitope‐tagged Glcent(s). (a) Western blot of *Giardia* cells expressing HA‐tagged Glcent1. (b) Immunofluorescence assays (IFAs) of interphase and dividing *Giardia* cells expressing HA‐tagged Glcent1 using rat anti‐HA and mouse anti‐acetylated‐α‐tubulin antibodies, a mitotic spindle marker. (c) IFA of anaphase and telophase *Giardia* cells expressing HA‐tagged Glcent1 using mouse anti‐HA and rat anti‐Glγ‐tubulin antibodies (a basal bodies marker). (d) Western blotting of *Giardia* cells expressing HA‐tagged Glcent2. (e) IFAs of interphase and dividing *Giardia* cells expressing HA‐tagged Glcent2 using rat anti‐HA and mouse anti‐acetylated‐α‐tubulin antibodies. (f) IFA of anaphase and telophase *Giardia* cells expressing the HA‐tagged Glcent2 using mouse anti‐HA and rat anti‐Glγ‐tubulin antibodies. (g) Western blotting of *Giardia* cells expressing HA‐tagged Glcent1 and myc‐tagged Glcent2. (h) IFAs of interphase and anaphase *Giardia* cells expressing HA‐tagged Glcent1 and myc‐tagged Glcent2 using rat anti‐HA and mouse anti‐myc antibodies. *Giardia* trophozoites carrying the vector plasmid(s) were included as controls (lane 1). GlPDI1 was monitored as a loading control for protein amount. For IFAs, the cells treated with primary antibodies were incubated with Alexa Fluor 555‐conjugated anti‐rat IgG and Alexa Fluor 488‐conjugated anti‐mouse IgG. Slides were mounted with the ProLong Gold Antifade Mountant with DAPI and then examined with a Zeiss LSM980 inverted confocal laser scanning microscope. The cell morphology is represented by differential interference contrast (DIC) images. Scale bars = 5 μm.

**Figure:**
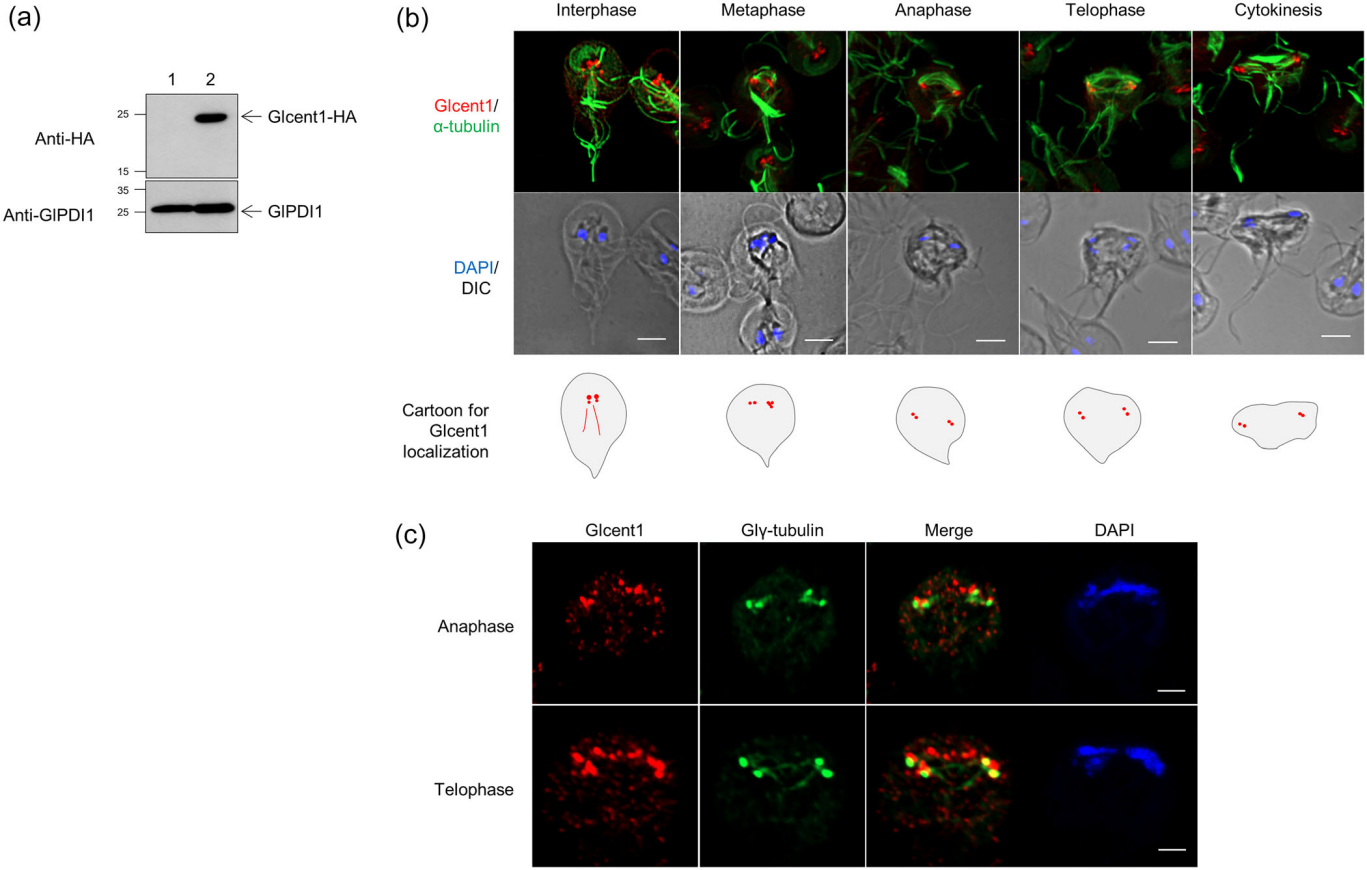

**Figure:**
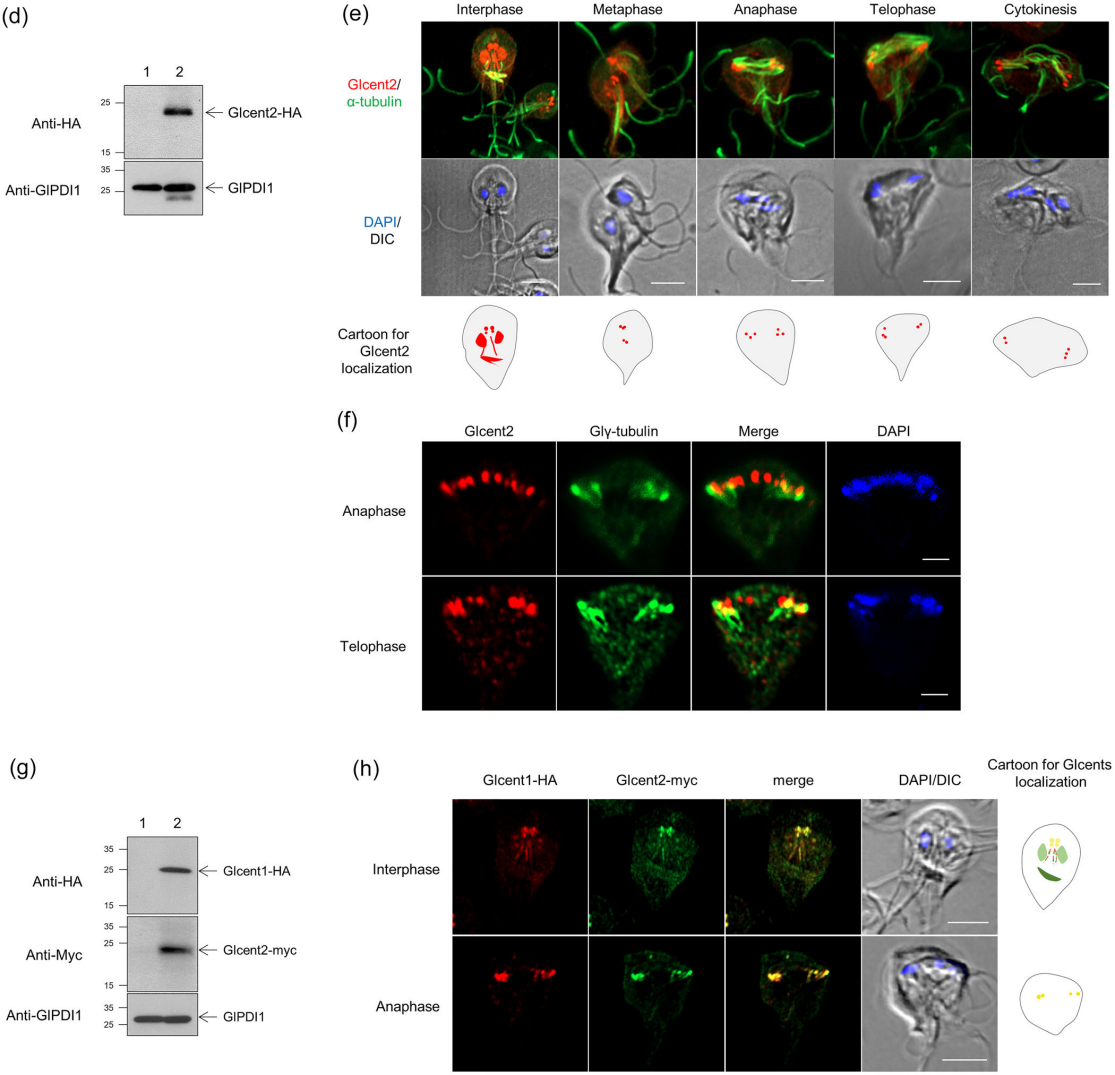

The expression of Glcent2‐HA (GL50803_104685) in *Giardia* cells harboring pGlcent2‐HA.NEO was demonstrated via western blot (Figure 1d). Interphase cells epigenetically expressing Glcent2 also demonstrated red fluorescence in the basal bodies and cytoplasmic axonemes of the posterolateral flagella (Figure 1e). These cells showed staining in the two nuclei and a median body. In dividing cells, Glcent2 was found at basal bodies, which were also stained with anti‐Glγ‐tubulin antibodies (Figure 1f). The MFI of Glcent2 in basal bodies was 211 (*n* = 23), and that was 141 in the nuclei (*n* = 25) in *Giardia* trophozoites.

To determine whether both Glcentrins are colocalized, the plasmid encoding the myc‐tagged Glcent2 was transfected into *Giardia* cells, making Glcent1‐HA. The resulting cells were used to examine the expression of both Glcentrins by western blotting using antibodies against the myc or HA epitope (Figure 1g). Upon double‐staining with these antibodies, basal bodies appeared as yellow fluorescence, indicating that Glcent1 and Glcent2 were colocalized at basal bodies in interphase and anaphase *Giardia* cells (Figure 1h). These observations suggested that both Glcents were expressed at the basal bodies through all cell cycle stages, and while only Glcent2 localized to the nuclei and median bodies of *Giardia* trophozoites.

### 3.2 The Amino‐Terminal Portion of Glcent2 Is Necessary for Its Nuclear Localization

**FIGURE 2:**
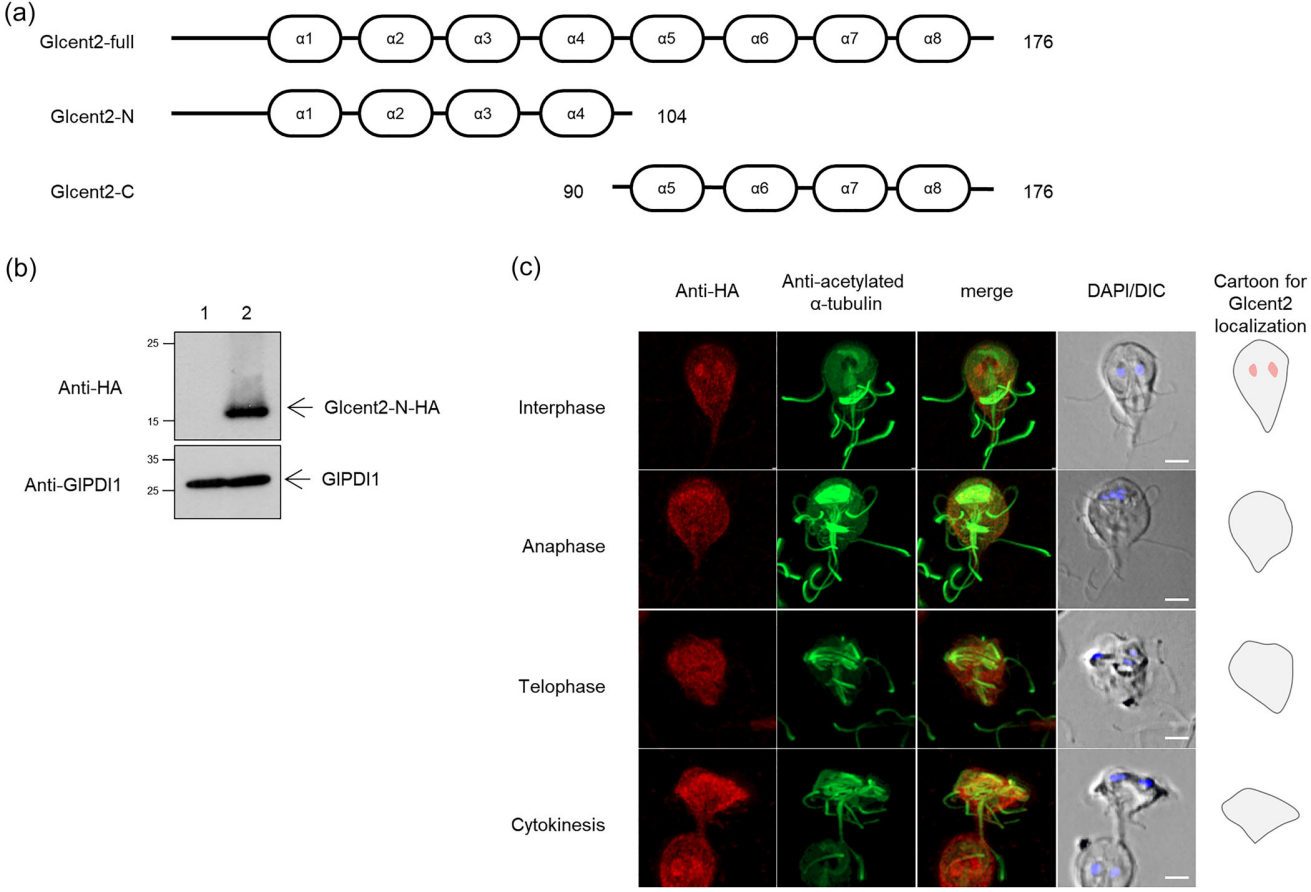
Expression and localization of truncated Glcent2 containing the amino‐terminal part of the protein. (a) Schematic diagram of two truncated Glcent2 proteins. These two proteins are expected to be expressed in an HA‐tagged form under their own promoter, P*glcent2*. Eight EF domains for Ca^2+^‐binding are indicated with serial numbers. Each construct is indicated with the amino acid residue numbers of Glcent2 included in the resulting protein. (b) Western blotting of *Giardia* cells expressing HA‐tagged Glcent2. *Giardia* trophozoites carrying the vector plasmid(s) were included as controls (lane 1). GlPDI1 was monitored as a loading control for protein amount. (c) Immunofluorescence assays of interphase and dividing *Giardia* cells expressing HA‐tagged Glcent2 using rat anti‐HA and mouse anti‐acetylated‐α‐tubulin antibodies. The cells treated with primary antibodies were incubated with Alexa Fluor 555‐conjugated anti‐rat IgG and Alexa Fluor 488‐conjugated anti‐mouse IgG. The cell morphology is represented by differential interference contrast (DIC) images. Scale bars = 2 μm.

A search within the Glcent2 ORF using a domain searching program (http://pfam.xfam.org/) indicated that it contained eight α‐helix domains (Figure 2a). To define the domains required for Glcent2 localization, we constructed two plasmids, pGlcent2‐N‐4H and pGlcent2‐C‐4H, which expressed the N‐terminal portion (α‐helix domains 1–4) and the C‐terminal part of Glcent2 (α‐helix domains 5–8), respectively. Expression of the truncated Glcent2 containing the N‐terminal portion was visualized in western blots with antibodies specific to the HA epitope (Figure 2b). On the contrary, *Giardia* harboring the empty plasmid pKS‐3HA.NEO did not demonstrate any immunoreactive bands. Cells expressing Glcent2 EF 1–4 demonstrated nuclear localization, while localization at the basal bodies was not clear (Figure 2c). Despite several attempts, we were unable to obtain cells expressing the carboxyl part of Glcent2 via transfection of the expression plasmid pGlcent2‐C‐4H into *Giardia* trophozoites (data not shown).

In the subsequent experiment, putative amino acid sequences of Glcent2 were examined for nuclear localization signal (NLS) using a web‐based prediction tool (NLS Mapper: https://nls-mapper.iab.keio.ac.jp/cgi-bin/NLS_Mapper_form.cgi). The search resulted in an NLS with a score of 2.5, which does not confirm nuclear localization. These data suggested that the N‐terminal α‐helical domain of Glcent2 mediates its nuclear localization. However, the nuclear localization of Glcent2 is not mediated by a typical NLS.

### 3.3 Glcent1 and Glcent2 Are Involved in the Division of *Giardia lamblia*

**FIGURE 3:**
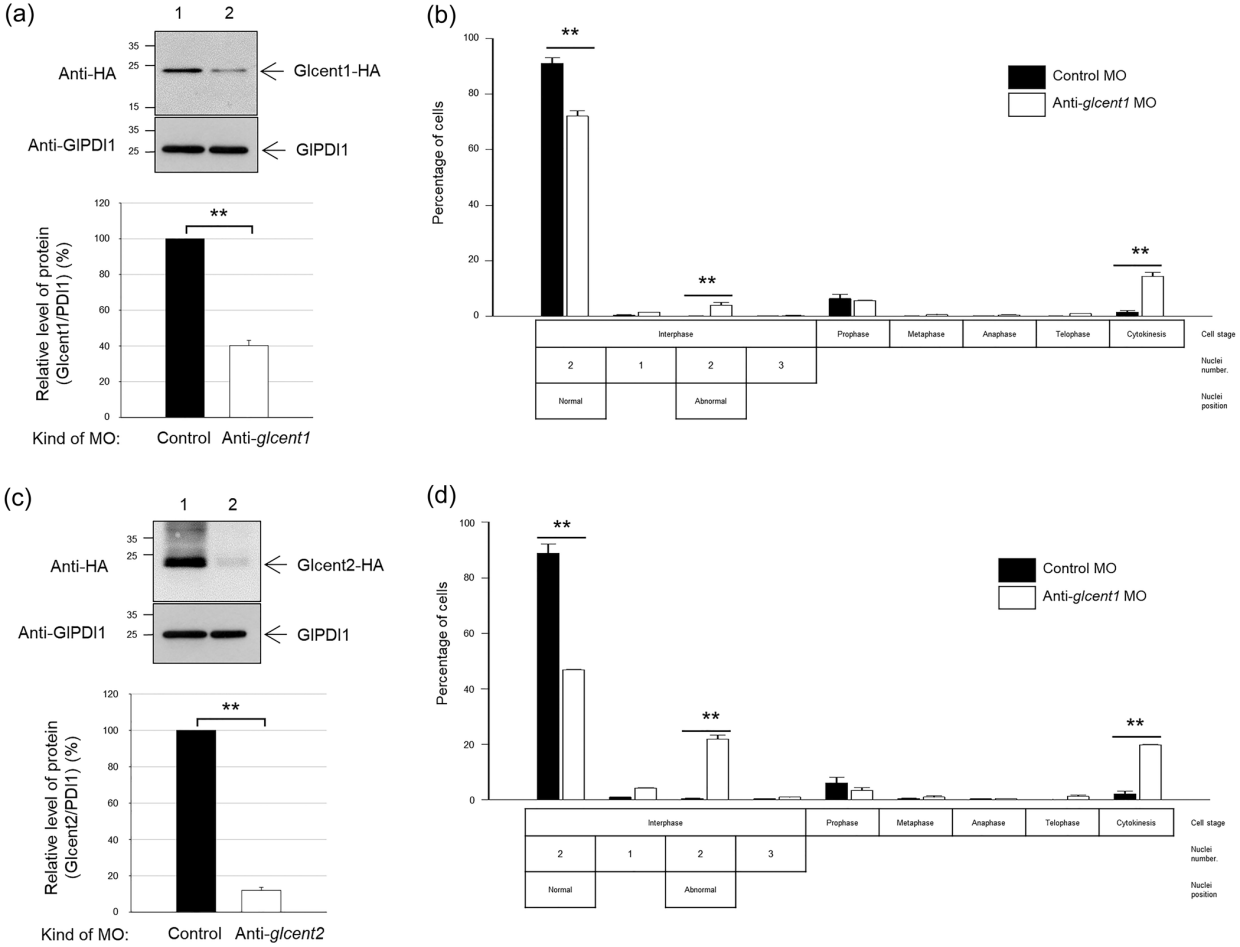
Effect of morpholino (MO)‐mediated Glcent knockdown on *Giardia* division. (a, b) *Giardia* cells expressing HA‐tagged Glcent1 were collected at 24 h after transfection with control (lane 1, closed bars) or anti‐*glcent1* (lane 2, open bars) MO. (a) Western blotting analysis of MO‐mediated Glcent1 knockdown (KD) in *Giardia* and a bar graph demonstrating the relative expression of HA‐tagged Glcent1 in cells treated with anti‐*glcent1* MO compared with that in the control cells. (b) Effect of MO‐mediated Glcent1 KD on the nuclear phenotypes and cell division of *G. lamblia*. (c, d) *Giardia* cells expressing HA‐tagged Glcent2 were collected at 24 h after transfection with control (lane 1, closed bars) or anti‐*glcent2* (lane 2, open bars) MO. (c) Western blotting analysis of MO‐mediated Glcent2 KD in *Giardia* and a bar graph demonstrating the relative expression of HA‐tagged Glcent2 in cells treated with anti‐*glcent2* MO compared with that in the control cells. (d) Effect of MO‐mediated Glcent2 KD on the nuclear phenotypes and cell division of *G. lamblia*. Data are presented as the mean of three independent experiments. **, *p* < 0.01.

To determine the function of Glcent1 in *Giardia*, we made an anti‐*glcent1* morpholino to inhibit the expression of *glcent1* transcripts (Table 1). A nonspecific morpholino oligomer was also designed and electroporated into *Giardia* cells harboring pGlCent1‐HA.NEO (Table 2). When these cells were collected at several timepoints (12–48 h), maximal suppression of Glcent1 expression was observed at 24 h posttransfection (data not shown). The level of Glcent1‐HA at 24 h posttransfection had diminished to 40% of that of the control cells (Figure 3a) (*p* = 0.00003). In contrast, GlPDI1 levels were equivalent among the cells incubated with control or anti‐*glcent1* morpholino.

The consequence of Glcent1 deficiency on the *Giardia* cell cycle was monitored in relation to the nuclear phenotypes and DNA status (Figure 3b). Cells with two correctly located nuclei diminished from 91% to 72%. The percentages of cells with incorrectly positioned nuclei increased to 4.1% in Glcent‐depleted cells from 0.1% of the control cells. The portion of cells at cytokinesis showed a significant increment (14%) in the Glcent1 knockdown cells (*p* = 0.001) compared to 1.5% of the control cells.

The influence of Glcent2 depletion on the division of *Giardia* was also measured (Figure 3c). The expression of Glcent2‐HA was reduced to 16% in cells transfected with an anti‐*glcent2* morpholino for 24 h. In cells treated with an anti‐*glcent2* morpholino, typical cells diminished to 47% from 89% of the control cells (Figure 3d). On the other side, the cells with abnormally positioned nuclei increased to 22% in the Glcent2‐depleted cells by comparison with the control cells (0.5%). The portion of *Giardia* cells undergoing cytokinesis increased to 20% from 2.2% of the control cells. Taken together, increased percentages of cells at cytokinesis under Glcent‐depleted conditions indicate an important role of both Glcents in the cell division of *G. lamblia*.

### 3.4 Depletion of Glcent1 or Glcent2 Causes Malformation of *Giardia lamblia*

**FIGURE 4:**
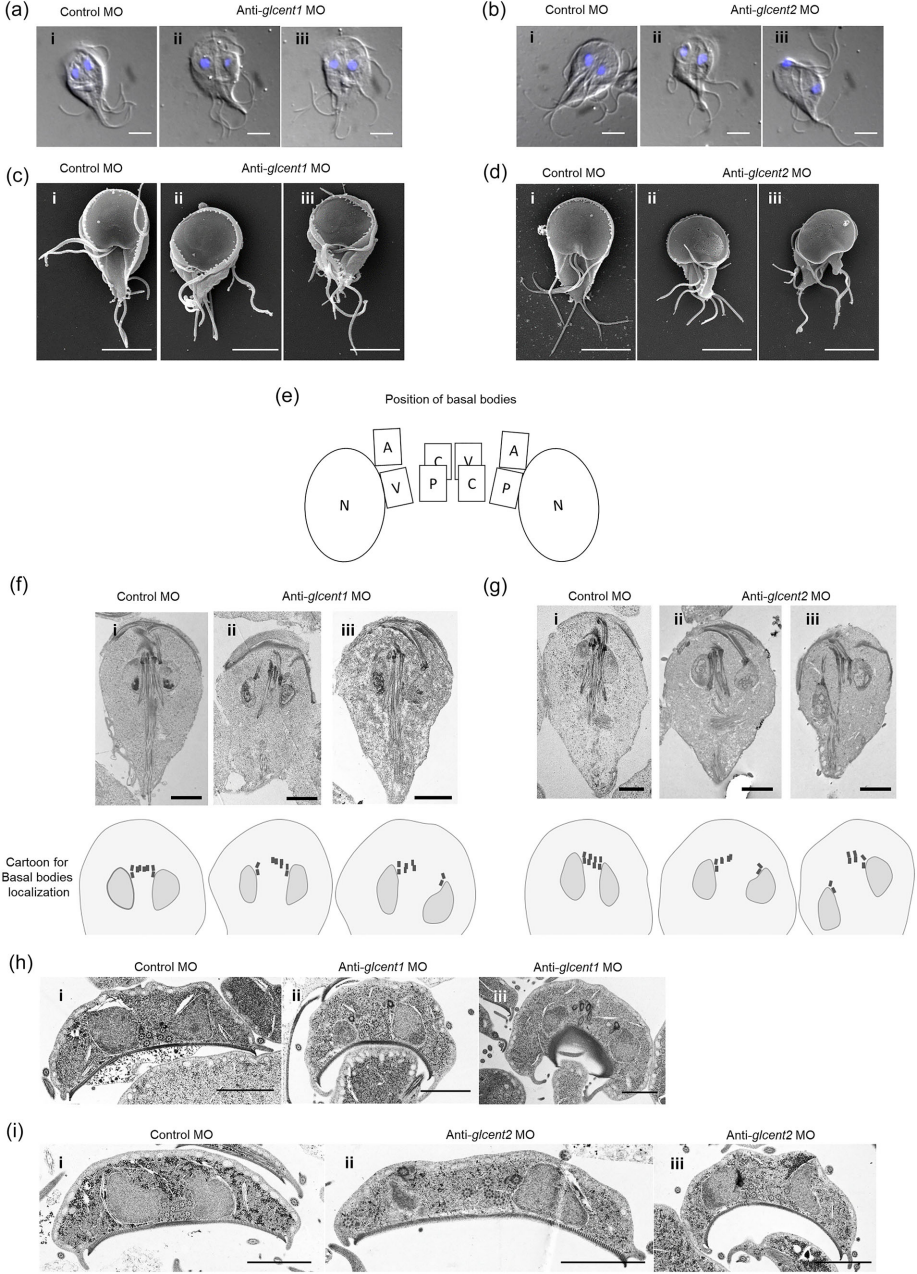
Effect of morpholino (MO)‐mediated Glcent knockdown on the morphology of *Giardia*. *Giardia* cells expressing HA‐tagged Glcent were collected at 24 h after transfection with control or anti‐*glcent* morpholino. Panel i presents *Giardia* cells treated with the control MO, whereas panels ii and iii demonstrate knockdown (KD) cells for Glcent1 or Glcent2. (a) Observation of Glcent1 KD cells by differential interference contrast (DIC). (b) Observation of Glcent2 KD cells by DIC. (c) Observation of Glcent1 KD cells by scanning electron microscopy (SEM). (d) Observation of Glcent2 KD cells by SEM. (e) Schematic diagram demonstrating the basal bodies for each pair of flagella. N indicates the nucleus, and basal bodies of caudal, anterior, posterolateral, and ventral flagella are annotated as C, A, P, and V, respectively. (f) Longitudinal transmission electron micrograph (TEM) images of Glcent1 KD cells. (g) Longitudinal TEM images of Glcent2 KD cells. (h) Transverse TEM images of Glcent1 KD cells. (i) Transverse TEM images of Glcent2 KD cells. Scale bars = 2 μm.

The increase in abnormally positioned nuclei among Glcent‐depleted cells drew our attention to their morphological changes. *Giardia* cells stained with DAPI were examined using differential image contrast (DIC) (Figure 4a, b). Compared to the cells transfected with a nonspecific morpholino (panel i), cells incubated with anti‐*glcent1* or anti‐*glcent2* morpholino (panels ii and iii) demonstrated deformed structures more frequently. The relative locations of the two nuclei changed. The positioning of the eight flagella was also altered in *glcent*‐depleted cells. In addition, deformation and incorrect positioning of the ventral discs were frequently observed in *glcent*‐depleted cells.

To more closely determine structural changes in the *Giardia* cytoskeleton caused by Glcent depletion, the cells were observed under a scanning electron microscope (Figure 4c, d). Both Glcent1 and Glcent2 depletion caused structural changes in the ventral discs. In particular, flanges surrounding the ventral discs were missing in Glcent2‐depleted cells. In addition, the protruding positions of the eight flagella were altered in Glcent‐depleted cells.

The intracellular structures of these cells, such as the nuclei and basal bodies of flagella, were scrutinized via transmission electron microscopy (TEM: Figure 4f–i). First, the Glcent‐depleted cells were longitudinally sectioned and observed using TEM (Figure 4f, g). Positions of the basal bodies of the four pairs of flagella are presented schematically in Figure 4e. Compared to the control cells (panel i), in which two nuclei were properly positioned and basal bodies were present between the two nuclei, Glcent‐depleted cells (panels ii and iii) showed irregular and frequently wider positioning of these structures. The degree of defects with respect to the relative position between nuclei and basal bodies was more severe in the Glcent2‐depleted than in the Glcent1‐depleted cells.

In addition, transverse sections of these cells were observed by TEM, providing information on the basal bodies, nuclei, and ventral discs (Figure 4h, i). In agreement with the longitudinal TEMs, the nuclei and cytoplasmic axonemes were present in more irregular and separated patterns in Glcent‐depleted cells (panels ii and iii) than in the control cells (panel i). The structure lining the ventral disc was more concave and degenerated in Glcent‐depleted cells. Interestingly, an absence or decrease of peripheral vesicles was frequently detected in Glcent knockdown cells. Observations of Glcent‐depleted cells via microscopy approaches indicated that both Glcents are involved in the formation of the nuclei, basal bodies, and ventral discs of *Giardia* trophozoites by maintaining the proper positioning of the basal bodies.

### 3.5 Glcent1 and Glcent2 Are Substrates of Small Ubiquitin‐Like Modifier (SUMO)

**FIGURE 5:**
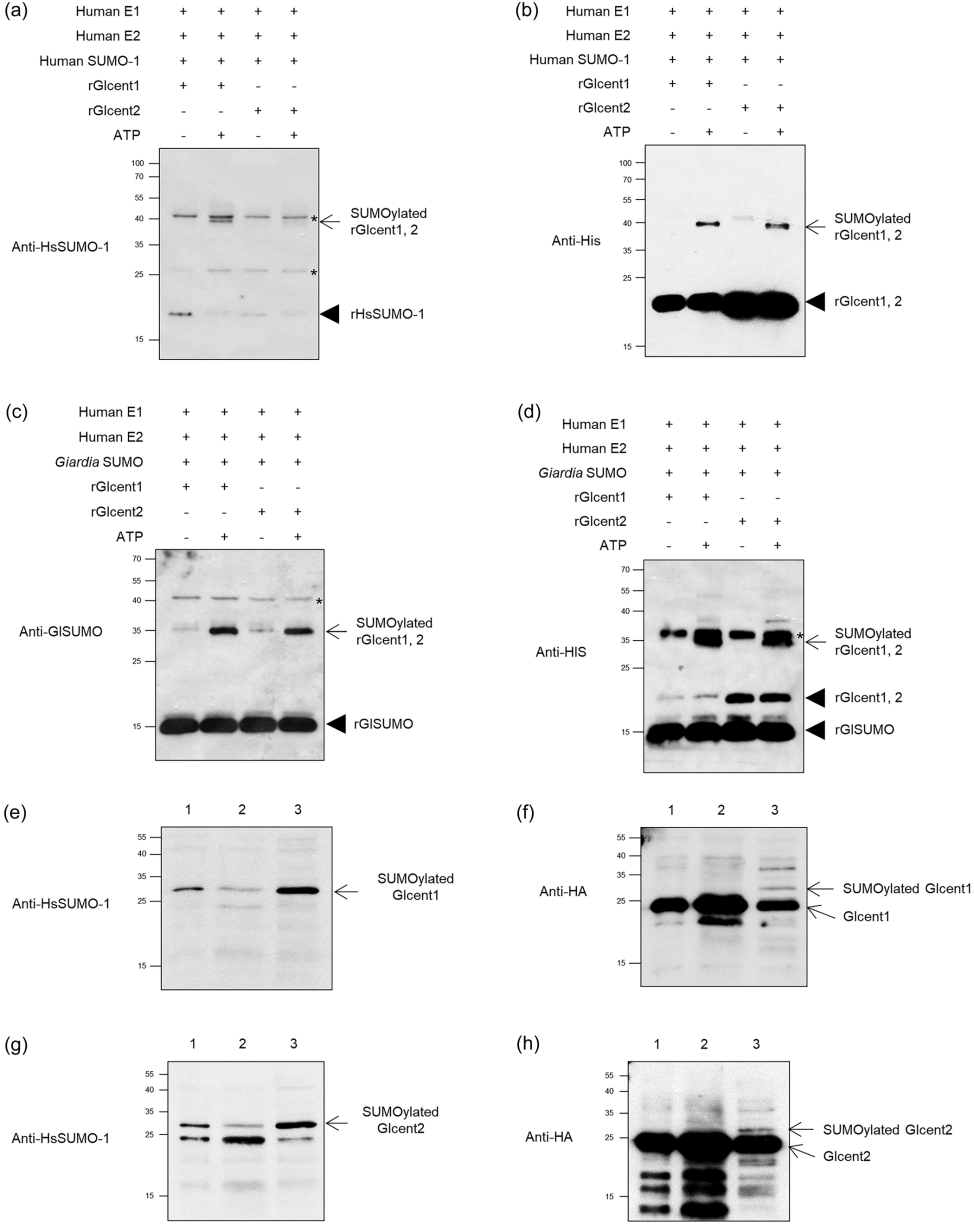
SUMOylation of Glcents. (a, b) In vitro SUMOylation of recombinant Glcent 1 (rGlcent1) and rGlcent2 using *Homo sapiens* SUMO‐1 (HsSUMO‐1). SUMOylation reactions with 5 μg rGlcents were performed using the components provided by the SUMOylation kit (BML‐UW8955) with or without ATP. The resulting reactions were separated by 12% SDS‐PAGE and transferred onto a polyvinylidene fluoride membrane. (a) Western blotting using polyclonal rabbit anti‐HsSUMO‐1 antibodies (1:1000 dilution). (b) Western blotting using monoclonal mouse anti‐histidine antibodies (1:10,000 dilution). (c, d) In vitro SUMOylation of rGlcent1 and rGlcent2 using *G. lamblia* SUMO (GlSUMO). (c) Western blotting using polyclonal rat anti‐GlSUMO antibodies (1:1000 dilution). (d) Western blotting using monoclonal mouse anti‐histidine antibodies (1:10,000 dilution). SUMOylated rGlcents are indicated by arrows, whereas unmodified SUMO or rGlcents are denoted with arrowheads. Nonspecific bands are indicated by asterisks. (e, f) Detection of SUMOylated Glcent1 by immunoprecipitation (IP) using the resin conjugated with SUMO‐interaction motif. Extracts of *Giardia* cells expressing HA‐tagged Glcent1 were incubated with the resin, and bound proteins were precipitated by centrifugation. (e) Western blot using polyclonal rabbit anti‐HsSUMO‐1 antibodies (1:1000 dilution). (f) Western blot using monoclonal mouse anti‐HA antibodies (1:1000 dilution). (g, h) Detection of SUMOylated Glcent2 by IP using the resin conjugated with the SUMO‐interaction motif. (g) Western blotting using polyclonal rabbit anti‐HsSUMO‐1 antibodies (1:1000 dilution). (f) Western blotting using monoclonal mouse anti‐HA antibodies (1:1000 dilution). The resulting IPs (lane 3) were analyzed by western blotting along with the cell extracts used for IP (lane 1) and flow‐through fraction (lane 2).

In mammals, centrin 2 is modified by SUMOylation, and this modification is required for its localization into the nucleus (Klein and Nigg 2009). In the following experiments, we examined whether Glcent1 or Glcent2 is posttranslationally modified by SUMO. First, two *Giardia* centrins were made as histidine‐tagged proteins in *E. coli* and purified. These recombinant proteins were used as a substrate for in vitro SUMOylation assay using human SUMO systems, namely human E1 and E2 and human SUMO‐1 (Figure 5a, b). Western blotting using anti‐HsSUMO‐1 antibodies revealed immunoreactive bands of approximately 17 kDa for the HsSUMO‐1 (Figure 5a). Western blotting of the reaction mixture was performed using anti‐histidine antibodies (Figure 5b). An immunoreactive band of 40 kDa appeared with the addition of ATP in the reaction, indicating the recombinant Glcent1 and Glcent2 modified by SUMOylation.

In the *Giardia* database, an open reading frame (ORF) is annotated as SUMO (GL50803_7760). This ORF was converted into a histidine‐tagged protein in *E. coli* and used for in vitro SUMOylation assay instead of HsSUMO‐1 (Figure 5c, d). Immunoreactive proteins of 35 kDa were detected in western blot analyses using antibodies against GlSUMO or histidine residues were GlSUMO‐conjugated proteins. These results indicated that both Glcent1 and Glcent2 could be substrates of SUMO in *Giardia*.

To determine whether SUMOylation of Glcent1 and Glcent2 occurs in vivo, *Giardia* cells expressing HA‐tagged Glcent were used to prepare extracts for immunoprecipitation (IP) with the resin conjugated with SUMO‐interaction motif (Figure 5e, f for Glcent1, and Figure 5g, h for Glcent2). Immunoreactive protein was more enriched in the IP fraction (lane 3) than in the loading (lane 1) or unbounded fraction (lane 2), as detected in western blotting using anti‐HsSUMO‐1 antibodies, and it appeared as a minor immunoreactive band in western blotting using antibodies specific to the HA epitope, which was speculated to be SUMOylated Glcents. These data suggested that both Glcent1 and Glcent2 are target substrates of SUMO in *Giardia*.

### 3.6 Inhibitor‐Mediated Blockage of SUMOylation Affects the Localization of Both Glcent1 and Glcent2

The finding that Glcentrins are modified by SUMO and that their depletion affects the morphology and binary fission of *Giardia* led us to investigate the function of SUMOylation in these processes. *G. lamblia* trophozoites were exposed to diverse amounts of ginkgolic acid (GA), which inhibits the SUMO E1 enzyme. The growth of *Giardia* trophozoites was inversely correlated with the GA concentration, with an IC_50_ of 80 M (Figure S1a).

*Giardia* cells were exposed to different concentrations of GA (60 to 120 μM) for 24 h, and the DNA content of the GA‐treated *Giardia* was monitored by flow cytometry (Figure S1b). The ratio of *Giardia* cells in the G2/M phase increased with GA concentration, and the DNA ploidy moved to the G2/M phase in almost all of the GA‐treated cells at the highest concentration (120 μM). The control cells included both G1/S‐phase (15%) and G2/M‐phase cells (85%). The fraction of G2/M‐phase cells increased to 96%, and the percentage of G1/S‐phase cells reduced to 4% upon the treatment with 120 μM GA. Next, *Giardia* cells were exposed to 0.24% DMSO or 120 μM GA for various periods (3 to 36 h) and then investigated for their DNA content (Figure S1c). The most pronounced G2/M phase arrest was observed in the cells exposed to 120 μM GA for 24 h, and this concentration was used for further studies (Figure S1d).

**FIGURE 6:**
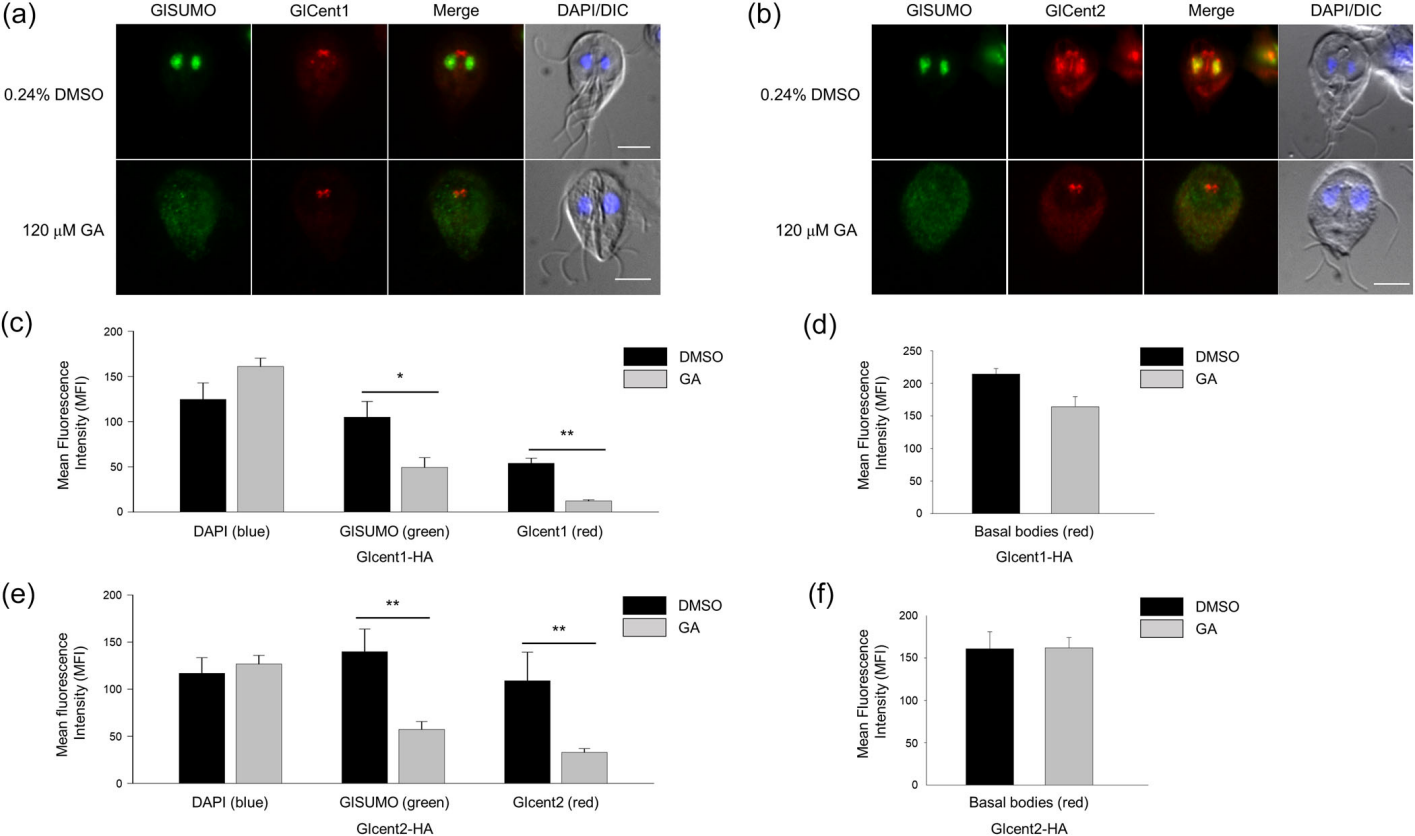
Effect of the SUMOylation inhibitor, ginkgolic acid (GA), on the localization of Glcents in *Giardia* trophozoites. *Giardia* cells expressing HA‐tagged Glcent1 (a) or Glcent2 (b) were treated with 120 μM GA for 24 h and subsequently examined by immunofluorescence assays (IFAs) using anti‐GlSUMO and anti‐HA antibodies. As a control, the same cells were treated with 0.24% DMSO instead of the inhibitor and examined by IFAs. Cell morphology is shown via differential interference contrast (DIC) images. Scale bars = 5 μm. The mean fluorescence intensities (MFIs) of basal bodies and nuclei in individual cells were quantified using ImageJ. (c) A bar graph of the nuclear MFIs of DAPI, GlSUMO, and Glcent1 in the HA‐tagged Glcent1 cells treated with GA compared with those treated with DMSO. (d) A bar graph of Glcent1 MFIs at the basal bodies in the HA‐tagged Glcent1 cells treated with GA compared with those in cells treated with DMSO. (e) A bar graph of the nuclear MFIs of DAPI, GlSUMO, and Glcent2 in the HA‐tagged Glcent2 cells treated with GA compared with those in cells treated with DMSO. (f) A bar graph of Glcent2 MFIs at the basal bodies in the HA‐tagged Glcent1 cells treated with GA compared with those in cells treated with DMSO. *0.01 < *p* < 0.05 and **p < 0.01.

We examined whether inhibition of SUMOylation affects the localization of Glcent1 using *Giardia* cells expressing HA‐tagged Glcent1. In order to detect SUMO, anti‐GlSUMO antibodies were used along with anti‐HA antibodies, which are employed for the detection of HA‐tagged Glcents. GlSUMO was observed in the nuclei of all cells treated with 0.24% DMSO, whereas this signal disappeared or was reduced in the cells treated with 120 μM GA for 24 h (Figure 6a, b). The green fluorescence derived from GlSUMO was significantly decreased to 49 MFI in the nuclei of GA‐treated cells (*n* = 6) from 109 MFI control cells (*n* = 3) (Figure 6c). In nuclei of the GA‐treated cells, red Glcent1 fluorescence was also dramatically reduced to 12 MFI compared to 54 MFI in the DMSO‐treated cells. GA treatment of cells expressing HA‐tagged Glcent1 decreased the MFI of basal bodies to 164 (*n* = 13) from 214 in the same cells treated with DMSO (*n* = 9), but without statistical significance (Figure 6d).

When the cells expressing HA‐tagged Glcent2 were treated with GA, nuclear GlSUMO and Glcent2 intensities decreased to 57 and 33 MFI (*n* = 9) in comparison with 140 and 109 MFI of the control cells (*n* = 3), respectively (Figure 6e). Glcent2 intensity at basal bodies was not altered by GA, remaining at 161–162 (*n* = 7–14) (Figure 6f). While IFAs indicated nuclear localization only for Glcent2, and not Glcent1, quantitative evaluation of HA‐tagged Glcent1 in nuclei suggested nuclear localization. These results indicated that both GlSUMO and Glcents are nuclear proteins, with GlSUMO depletion causing defects in the nuclear localization of both Glcents.

### 3.7 Inhibitor‐Mediated Blockage of SUMOylation Affects the Division and Morphology of *G. lamblia*

**FIGURE 7:**
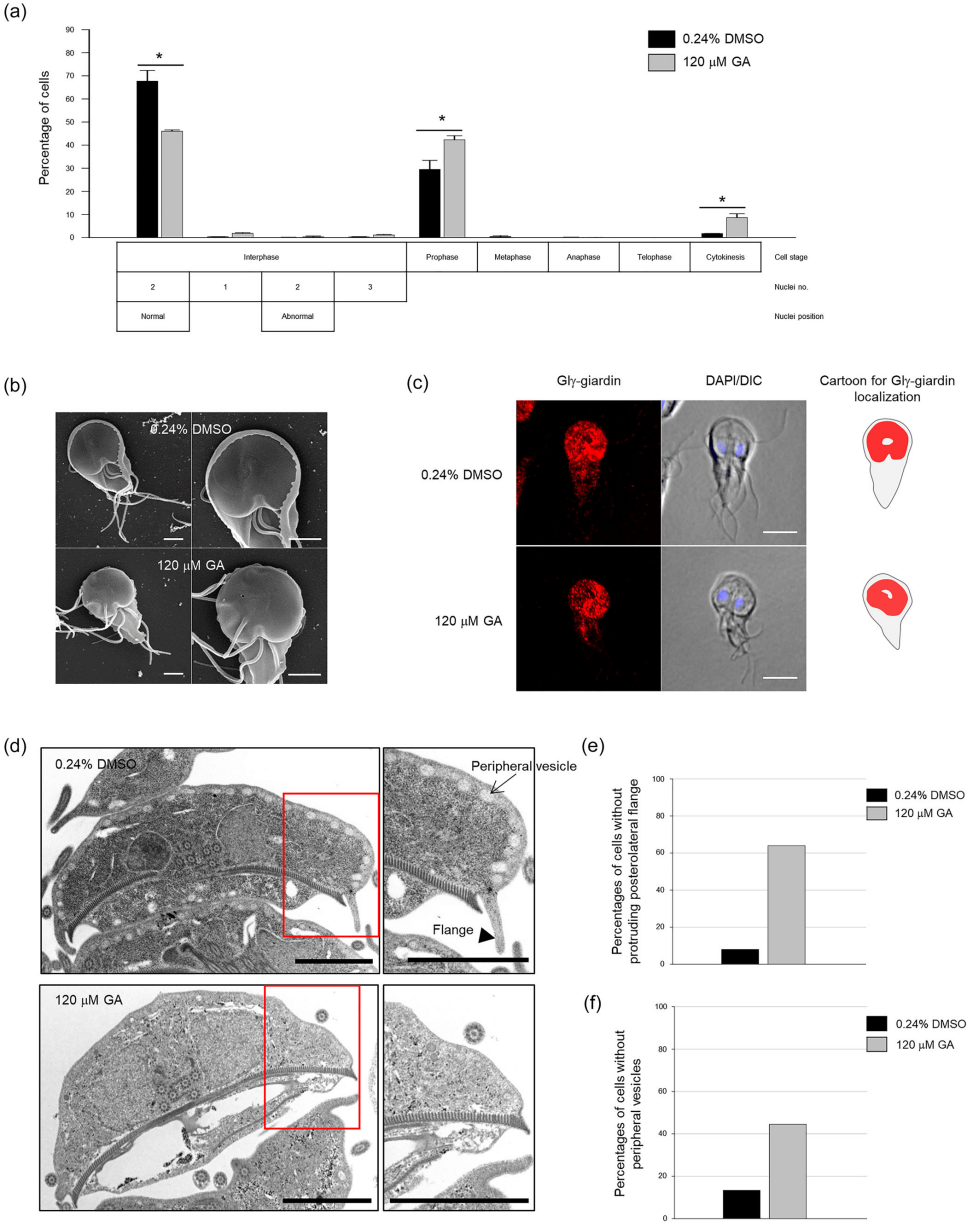
Effects of the SUMOylation inhibitor, ginkgolic acid (GA), on the cell division and morphology of *Giardia lamblia*. *Giardia* trophozoites were treated with 120 μM GA for 24 h (gray bars), and then stained with 10% Giemsa for division phenotype, or fixed for scanning electron microscopy (SEM), immunofluorescence assays (IFAs), and transmission electron microscopy (TEM). As a control, the same cells were treated with 0.24% DMSO instead of the inhibitor (closed bars). (a) Effect of GA on the nuclear phenotypes and cell division of *G. lamblia*. The asterisks indicate statistically significant differences at *, 0.01 < *p* < 0.05. (b) SEM images of the control (treated with 0.24% DMSO) and GA‐treated cells. Enlarged images are also presented to enable the comparison of the flanges surrounding ventral discs between these cells. Scale bars = 2 μm. (c) IFAs of the control and GA‐treated cells using rat anti‐Glγ‐giardin antibodies. The cell morphology is shown in differential interference contrast (DIC) images. Scale bars = 5 μm. (d) TEM images of the control and GA‐treated cells. The portions of TEM images indicated with red‐lined boxes are presented as enlarged panels for observation of the formation of flanges (arrowhead) and peripheral vesicles (arrow). Scale bars = 2 μm. (e) Bar graph showing the percentages of cells with protruding flanges among the control and GA‐treated cells. (f) Bar graph comparing the percentages of cells with peripheral vesicles between the control and GA‐treated cells.

To observe the effects of inhibiting SUMOylation on Giardia cell division, Giardia trophozoites were exposed to 120 µM GA for 24 h and stained with Giemsa. Nuclear position was evaluated and scored with respect to the stages of mitosis and cytokinesis in case of dividing cells (Figure 7a). The percentage of interphase cells with normal nuclear position decreased to 46% from 68% (*p* = 0.02), whereas the cells showing DNA condensation at prophase changed to 42% from 29% in control *Giardia* (*p* = 0.04). In addition, the portion of cells at cytokinesis increased from 1.7% to 8.6% under SUMOylation‐inhibited conditions (*p* = 0.04).

The morphology of GA‐treated *Giardia* trophozoites was examined using SEM (Figure 7b). Compared to the control cells incubated with 0.24% DMSO, the distortion of the cells' bodies and alteration of the ventral discs were distinct. Notably, the flanges surrounding ventral discs disappeared more frequently in the GA‐treated cells. In addition, flagellar biogenesis was affected in the inhibitor‐treated cells with respect to their position and length.

Structural distortion of the ventral discs was examined by IFA of the GA‐treated cells using antibodies against *G. lamblia* γ‐giardin comprising *Giardia* adhesive disc (Kim and Park 2019b) (Figure 7c). While the control cells showed normal formation of the ventral groove located at a posterior part of the adhesive disc showing convex curvature, the GA‐treated cells lost shrinkage in the ventral disc, leading to the formation of a round shape.

The GA‐treated cells were observed for cytoplasmic axonemes of the flagella and nuclei using TEM (Figure 7d). Most of all, the transverse sections of cells showed that the position of the two nuclei and axonemes was not significantly altered by the GA treatment. Interestingly, the loss of flanges of the ventral disc was distinct in GA‐treated cells (63%) compared to 8% of the control cells (Figure 7e). In the case of GA‐exposed cells, *Giardia* with a decreased number of peripheral vesicles were more frequently observed (44%) than the control cells (13%) (Figure 7f). These results indicated that cytoskeletal proteins, comprising the flagella and adhesive discs, could be substrates of SUMO in *Giardia*.

### 3.8 CRISPRI‐Mediated Knockdown of Ubc9 Affects the Division and Morphology of *G. lamblia*

**FIGURE 8:**
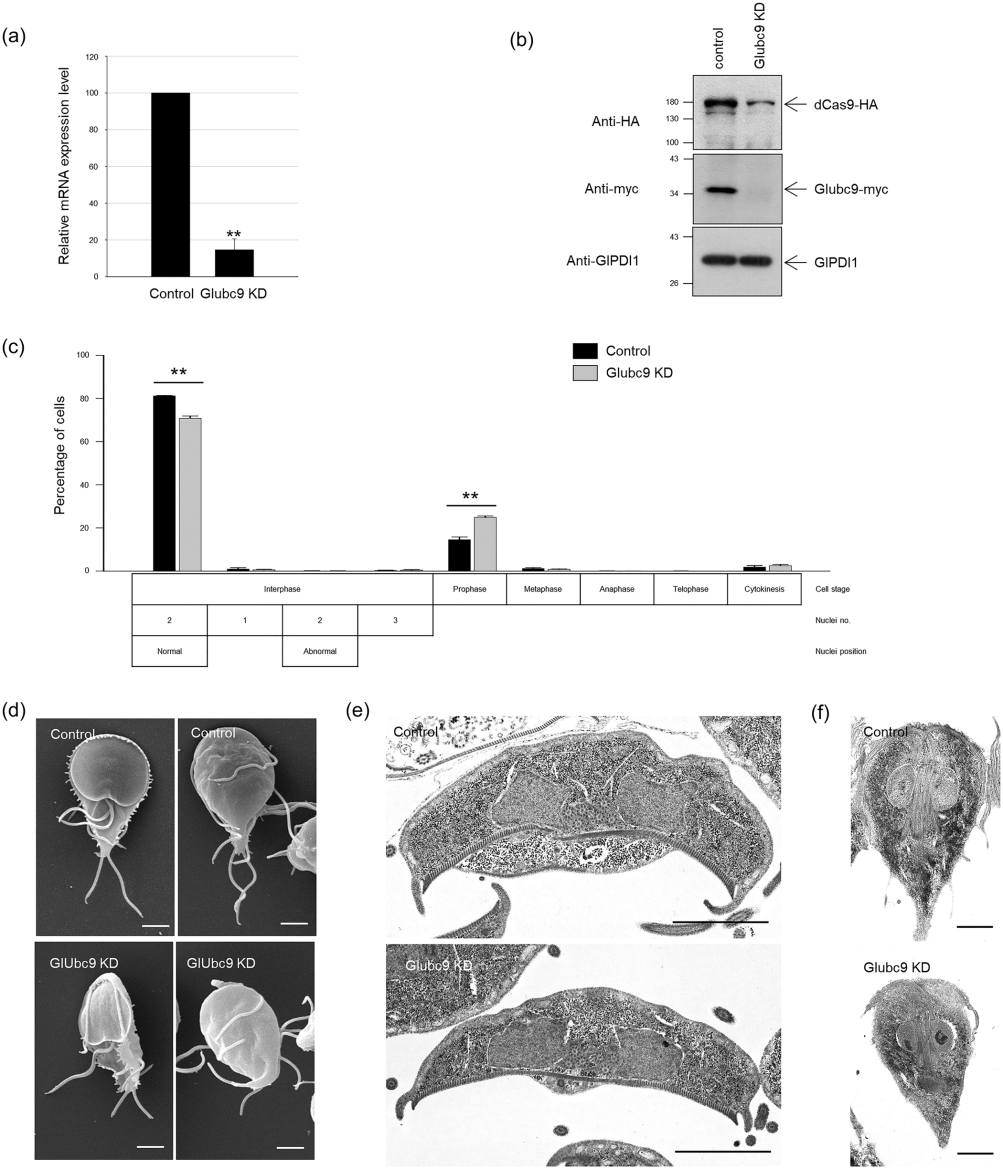
CRISPRi‐mediated knockdown (KD) of *Giardia lamblia* ubc9, Glubc9, and its effect on cell division and morphology of *G. lamblia*. (a) Quantitative real‐time PCR for *glubc9* transcripts. (b) Western blotting of the control and Glubc9 KD cells using anti‐HA (for dCas9 expression), anti‐myc (for Glubc9 expression), and anti‐GlPDI1 antibodies as a loading control. (c) Effect of Glubc9 KD on cell division. Both control and Glubc9 KD cells were stained with 10% Giemsa for the observation of nuclear phenotype and cell division phases. (d) Dorsal and ventral views of control and Glubc9 KD cells by scanning electron microscopy. (e) Transverse transmission electron micrograph (TEM) images of the control and Glubc9 KD cells. (f) Longitudinal TEM images of the control and Glubc9 KD cells. Scale bars = 2 μm. **p < 0.01.

The role of SUMOylation was also determined in *Giardia* cells with reduced SUMOylation activity through the knockdown of Ubc9, a SUMO‐conjugating E2 enzyme. *Giardia* cells expressing myc‐tagged Glubc9 were transfected with a dead Cas9 (dCas9)‐mediated knockdown system along with one of the two guide RNAs (gRNAs), which were synthesized to interfere with the transcription of the *glubc9* gene at +29, or +43 relative to the start codon of Glubc9 (Table 1). As a control, a random oligonucleotide sequence was cloned into a dCas9‐expressing plasmid and transfected into *Giardia*, and the resulting transfectants were monitored to determine the transcriptional level of *glubc9* mRNA. The gRNAs annealed at +29 did not affect the production of *glubc9* transcript, whereas those annealed at +43 caused a decrease in *glubc9* mRNA (data not shown). In cells expressing +43 gRNA for *glubc9*, the transcript level diminished to 15% of that in the control cells (Figure 8a). We used this transfectant as the Glubc9 knockdown (KD) cells. Cell extracts of Glubc9 KD and control cells were analyzed by western blot analysis using antibodies specific to the HA epitope to confirm dCas9 expression, and antibodies specific to the myc epitope were used to demonstrate the reduced level of Glubc9 protein in Glubc9 KD cells (Figure 8b). The level of GlPDI1 was determined in the same extracts as a loading control for protein amount.

The resulting Glubc9 KD cells were stained with Giemsa and measured for the nuclear morphology as well as the stages of mitosis and cytokinesis (Figure 8c). The percentage of interphase cells with normal morphology decreased to 71% from 81% (*p* = 0.0004), whereas the prophase cells increased to 25% from 15% of the control cells (*p* = 0.0005).

SEM images of Glubc9 KD cells indicated distortion of the cell body, flagella, and ventral discs (Figure 8d). Flanges lining ventral discs were disintegrated in the knockdown cells. TEM images indicated that the changes in cytoplasmic axonemes were not distinct, but the frequency of cells losing flanges or degenerated flanges increased with Glubc9 depletion (from 0% to 20%) (Figure 8e). However, the percentages of cells affected by the formation of peripheral vesicles did not change with Glubc9 expression. Longitudinal TEMs did not reveal any significant alterations in the positions of nuclei and axonemes between the control and the Glubc9 KD cells (Figure 8f). Deficiency of Glubc9 led to an increase in the number of prophase cells and induced morphological changes in structures such as flagella, cell bodies, ventral discs, and flanges. However, no positional change of the basal bodies was observed in these cells.

### 3.9 CRISPRI‐Mediated Knockdown of Ubc9 Affects the Localization of Glcent2

**FIGURE 9:**
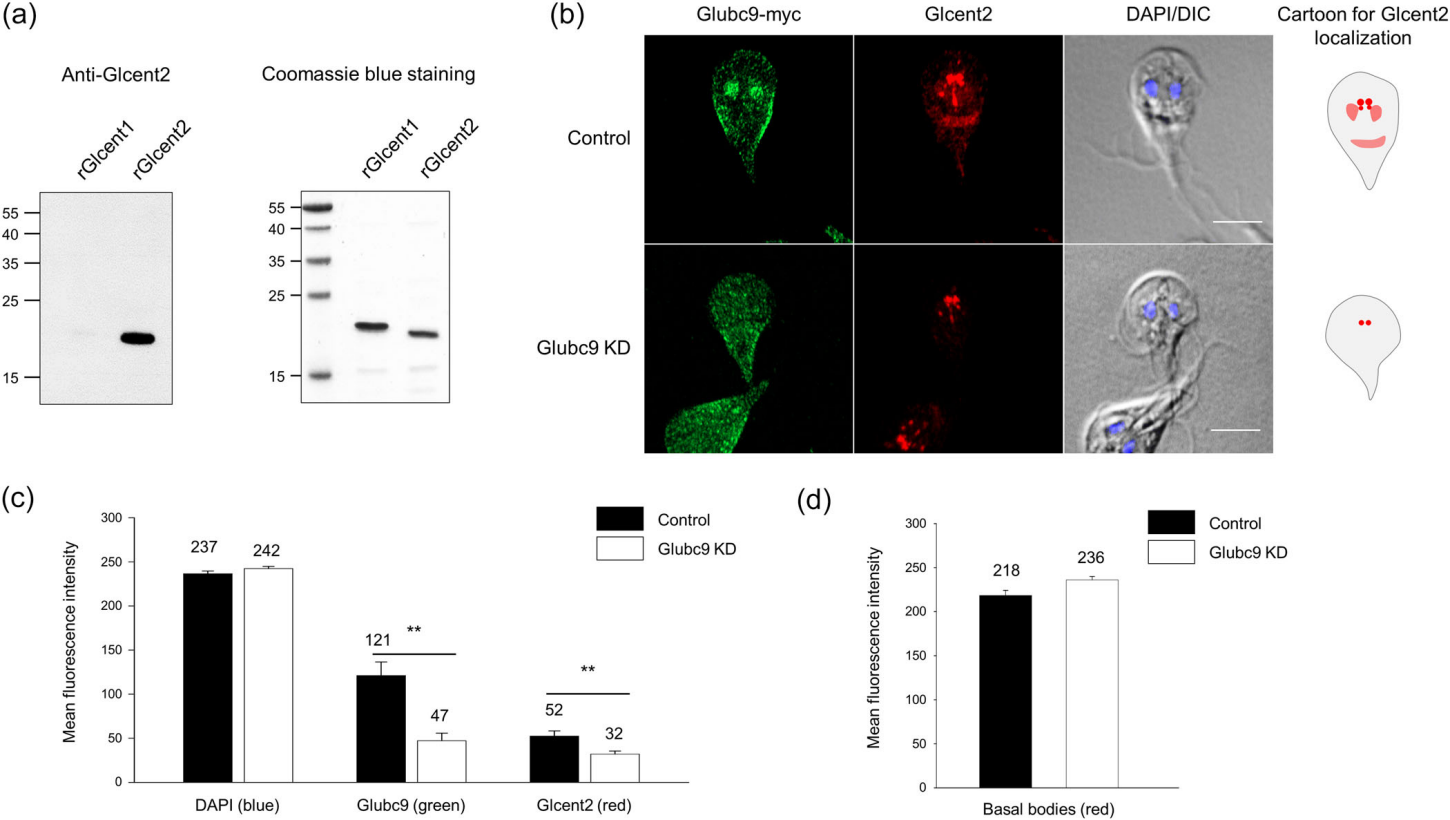
Localization of Glcent2 in Glubc9 knockdown (KD) cells. (a) Specificity of anti‐Glcent2 antibodies. Recombinant Glcent1 (rGlcent1) and rGlcent2 proteins were reacted with polyclonal antibodies against rGlcent2. Both proteins were also visualized by Coomassie blue staining. (b) Immunofluorescence assays of the control and Glubc9 KD cells using anti‐myc and anti‐rGlcent2 antibodies. *Giardia* cells expressing myc‐tagged Glubc9, HA‐tagged dCas9, but with a random guide RNA (gRNA) instead of gRNA for *glubc9* serve as the control cells. The cells treated with primary antibodies were incubated with Alexa Fluor 488‐conjugated anti‐mouse IgG and Alexa Fluor 568‐conjugated anti‐rat IgG. The cell morphology is represented by differential interference contrast (DIC) images. Scale bars = 5 μm. The mean fluorescence intensities (MFIs) of basal bodies and nuclei were measured using ImageJ. (c) A bar graph of the Glcent2 MFIs in basal bodies in Glubc9 KD cells compared with those in the control cells. (d) A bar graph of the nuclear MFIs of DAPI, Glubc9, and Glcent2 in Glubc9 KD cells compared with those in the control cells. **p < 0.01.

As described previously, *Giardia* cells treated with GA demonstrated alterations in the localization of both Glcentrins (Figure 6). In particular, the localization of Glcent2 in the nuclei and basal bodies decreased in SUMO inhibitor‐treated cells (Figure 6b). In this study, we examined whether Glubc9 depletion also causes defects in Glcent2 localization using the Glubc9 KD cells and Glcent2‐specific polyclonal antibodies (Figure 9). The specificity of antibodies raised against recombinant Glcent2 protein, which did not react with recombinant Glcent1 protein, was confirmed using western blotting (Figure 9a). Both the control and Glubc9 KD cells were reacted with antibodies specific to the myc epitope to determine their Glubc9 expression or antibodies against Glcent2 to observe the localization and expression of Glcent2 by IFAs (Figure 9b). Although the nuclear and peripheral expression of Glubc9 was observed in control cells, its expression was not distinct in the Glubc9 KD cells. Control cells showed Glcent2 expression at the basal bodies, nuclei, and median bodies. The MFI of Glcent2 in the basal bodies was 218 in the control cells (*n* = 5) and 236 in Glubc9 KD cells (*n* = 4) (Figure 9d). However, the nuclear amount of both Glubc9 and Glcent2 was statistically decreased to 47 and 32 MFI (*n* = 8) in comparison with 121 and 52 MFI of the control cells (*n* = 8), respectively (Figure 9c). Especially, the effect on their localization at nuclei and median bodies was more drastic than that at the basal bodies. The phenotypes observed in Glubc9 KD cells with respect to Glcent2 localization were comparable to those in the GA‐treated cells (Figure 6), confirming that SUMOylation of Glcent2 is involved in its nuclear localization in *Giardia*.

## 4 Discussion

The basal body serves as the microtubule‐organizing center (MTOC), which functions as an important organelle for flagella and spindle pole formation in *Giardia* (Elmendorf et al. 2003). Two centrin proteins, *G. lamlbia* centrin 1 (Glcent1; GL50803_6744) and Glcent2 (GL50803_104685), have been identified by genomic and proteomic analyses of the basal body (Lauwaet et al. 2011). The number of centrin genes has been discovered to vary among eukaryotes. Unicellular eukaryotes, such as *Saccharomyces cerevisiae* and *Chlamydomonas reinhardtii*, possess one centrin gene (Baum et al. 1986; Huang et al. 1988), whereas higher eukaryotes usually have several genes. For example, there are three *Homo sapiens* centrin (Hscent) genes (Lee and Huang 1993; Errabolu et al. 1994; Middendorp et al. 1997). Unicellular organisms with cilia have a large number of centrin genes, with 50 genes in *Paramecium tetraurelia*, 22 genes in *P. caudatum*, and 15 genes in *Tetrahymena thermophila* (Aubusson‐Fleury et al. 2017). The sequence identity between the two Glcentrins was 61%. Alignment of the amino acid sequences of Glcents and Hscents revealed that Glcent1 demonstrated sequence identity of 72% and 75% with Hscent1 and Hscent2, respectively, and that Glcent2 had 64% identity with Hscent3.

Morpholino‐mediated knockdown (KD) experiments were performed to differentiate the functions of these two Glcents. The result clearly indicated that the depletion of both Glcentrins using a specific morpholino resulted in an increase in cells undergoing cytokinesis and cells with mis‐positioned nuclei (Figure 3). Fewer KD cells were attached to culture tubes compared to control cells, indicating an abnormality of *Giardia* cytoskeletal structures in Glcent‐deficient cells as observed in the experiments using various microscopes (Figure 4). In *Giardia*, basal bodies act as MTOC (Elmendorf et al. 2003). The eight basal bodies are present closer to the anterior part of the cell between the two nuclei of *Giardia* trophozoites (Figure 1), from which the flagella are nucleated, and organized into four bilaterally symmetrical pairs: anterior, posterolateral, ventral, and caudal flagella (Meng et al. 1996; Corrêa et al. 2004). Interestingly, axonemes for each pair of flagella are associated with cytoskeletal structures (Dawson and House 2010; McInally and Dawson 2016). The marginal plates of adhesive discs are connected with the anterior axonemes, whereas the funis microtubules are associated with the caudal flagella. In addition, posterolateral and ventral axonemes are linked to the electron‐dense material and the fin‐like structures of *Giardia* trophozoites, respectively. The ventral disc nucleates from the caudal axonemes, forming a right‐handed spiral array (Hagen et al. 2011; Schwartz et al. 2012). The altered position of basal bodies caused by the depletion of Glcents changes the position of flagellar axonemes, which results in defects in the formation of ventral discs, marginal plates, and funis, thereby affecting the overall morphology of *Giardia* trophozoites.

Similar to the centrosomes in human cells, *Giardia* basal bodies are present at the spindle poles during mitosis (Figure 1) (Benchimol 2005; Davids et al. 2008; Kim et al. 2014). TEM analysis of longitudinal sections showed that the basal bodies associated with the nuclei were shifted to the left or right in Glcent‐deficient cells, whereas the basal bodies were aligned side by side in the control cells (Figure 4e, f). *Giardia* nuclei were connected to the anterior and the posterolateral axonemes on the right side, and linked to the anterior and the ventral axonemes on the left side. Glcentrin was found between the axonemes and nuclei via immune‐TEM using immunogold conjugated anti‐Hscentrin antibodies, suggesting that Glcentrin links the basal bodies and nuclei (Benchimol 2005). *Chlamydomonas* centrin was found between the nucleus and basal bodies in connectors and stellate fibers, maintaining their structures (Wingfield and Lechtreck 2018).

During *Giardia* mitosis, both Glentrins were observed at the spindle pole during nuclear migration, and then moved to the nuclear margins as the spindles elongated (Figure 1) (Nohýnková et al. 2006). Previous reports proposed that movement of basal bodies and nuclei is a mutually regulated process mediated by the centrin‐dependent adhesion of basal bodies to the nuclear envelopes (Nohýnková et al. 2006; Benchimol 2005; McInally and Dawson 2016;). Our results support the notion that the nuclei are interconnected to the basal bodies in a Glcent‐mediated way and that they migrate together during mitosis. Interestingly, the effects of Glcent depletion were more severe in Glcent2 KD cells than in Glcent1 KD cells. However, evidence is not sufficient to confirm a greater importance of Glcent2 than Glcent1, as the degree of depletion by a specific morpholino was more deleterious in Glcent2 KD than in Glcent1 KD cells (Figure 3a, c).

Glcentrins were mainly expressed at the basal bodies and also presented posterolateral axonemes in interphase cells (Figure 1). Remarkably, Glcent2 was also found in the median bodies and nuclei of *Giardia* trophozoites. While the localization of Glcentrin at median bodies has been previously reported (Meng et al. 1996; Corrêa et al. 2004), the nuclear expression of Glcent2 is reported here for the first time (Figure 1e, f, h). Ca^2+^‐binding EF motif of Glcent2 (Figure 2) and its SUMOylation affected its nuclear localization (Figures 6 and 9, respectively). In humans, Hscent1 is abundant in male germ cells and sperm (Avidor‐Reiss et al. 2019), while Hscent2 and Hscent3 are expressed in all somatic cells (Moretti et al. 2023). *Trypanosoma brucei* centrin 2 was found at a bi‐lobed structure near the Golgi apparatus (He et al. 2005), suggesting that centrins have different functions depending on their localization. In addition to the four Ca^2+^‐binding EF‐hand motifs, Hscent2 has an additional C‐terminal domain that interacts with the xeroderma pigmentosum complementation group C protein. Formation of this complex is required for its nuclear localization and participation in NER (Klein and Nigg 2009; Nishi et al. 2013). Our results showed that the N‐terminal half‐motif of Glcent2 is essential for its nuclear localization (Figure 2). Lack of a plausible NLS in the amino acid sequences of Glcent2 led us to search for other factors determining the nuclear localization of Glcent2, including interacting proteins with Glcent2.

SUMOylation of Hscent2 protein is also critical for its function in NER. Disruption of the SUMOylation pathway triggered defects in the nuclear localization of Hscent2, whereas its centrosomal recruitment remained unaffected (Klein and Nigg 2009). Therefore, we examined whether modification of Glcents affects their function. *G. lamblia* has genes encoding one SUMO (GLSUMO: GL50803_7760), three SUMO‐specific enzymatic proteins (E1 activating enzyme: Uba2, GL50803_6288; E2 conjugation enzyme: Ubc9, GL50803_24068; and SUMO‐specific protease: GL50803_16438), and two putative SUMO‐related E3 ligases (GL50803_11930 and GI50803_10261) (Vranych et al. 2012; Castellanos et al. 2020). GlSUMO‐deficient *Giardia* cells were retarded in proliferation, showing an arrest at the G1/S phase. Twenty‐seven proteins were proposed as GlSUMO substrates, among which α‐tubulin was confirmed as a SUMO substrate via immunoprecipitation (IP) (Di Genova et al. 2016). Arginine deiminase (ADI) is another known SUMO substrate in *Giardia*. SUMOylation of Lys101 of ADI facilitates its nuclear localization and results in a decrease of cyst wall protein (CWP) expression during encystation (Touz et al. 2008) via modification of histones at the *cwp* promoters (Vranych et al. 2014). Our results for Glcents extend the list of validated GlSUMO substrates (Figure 5). Using the GPS‐SUMO 1.0 tool [https://sumo.biocuckoo.cn/download.php], the SUMO target residues in Glcentrins were predicted to be lysine 53 of Glcent1 and lysine 33 and 68 of Glcent2. We observed that SUMO inhibition using GA or CRISPRi‐mediated Glubc9 KD influenced Glcentrin expression and the nuclear localization of Glcent2 (Figures 6 and 9, respectively).

Similar to the effects of Glcents‐depletion, we observed deformations of the ventral disc, changes in flagellar length, and shortening of the flange in SUMO‐deficient cells, such as in GA‐treated and CRISPRi‐mediated Glubc9 KD cells. These observations suggest that the components involved in the formation of these structures may be modified by SUMOylation for their proper functions. In contrast, the position of basal bodies was not significantly affected in these SUMO‐deficient cells (Figures 7 and 8). The effect of Glcentrin depletion in the formation of the basal body was expected to be more drastic than the result of a decrease in SUMOylated Glcentrins. More experiments should be performed to determine the exact role of SUMOylated Glcent in *Giardia* cell cycle control and morphogenesis of *Giardia* cells. Since SUMOylation acts on diverse targets, causing pleiotropic effects in *Giardia* cells, various phenotypes could be expected for Glcent depletion, GlSUMO inhibition, and Glubc9 depletion.

In addition to SUMOylation, Hscentrin is phosphorylated by a cAMP‐dependent kinase (PKA) upon the separation of centrosomes at prophase (Lutz et al. 2001), raising the possibility that Glcents are modified through other processes, such as phosphorylation. In addition to canonical proteins (e.g., delta‐tubulin, centrin, and epsilon‐tubulin), cell cycle‐related proteins (NIMA‐related kinase, aurora kinase, and polo‐like kinase) and proteins involved in signaling pathways (protein kinase R, extracellular signal‐regulated kinase 1, PKA, and protein phosphatase 2A) were found in the basal bodies of *Giardia* cells (Meng et al. 1996; Corrêa et al. 2004; Di Genova et al. 2016; Davids et al. 2008).

Reduction or disappearance of peripheral vesicles (PVs) was distinct in cells treated with the GA inhibitor (Figure 7d). *Giardia* PVs are small vacuoles located beneath the plasma membrane and play a role in the endocytosis of *Giardia* (Kattenbach et al. 1991). PVs are a part of interconnected networks along with the endoplasmic reticulum (ER), and their contact sites may be implicated in the transport of designated cargo from PVs to the ER (Abodeely et al. 2009). In *Giardia*, the endocytic machinery is associated with clathrin heavy chain (GL50803_102108), a dynamin‐related protein (GL50803_14373), and the clathrin adaptor protein 2 complex heterotetramer (GL50803_5328) (Gaechter et al. 2008; Rivero et al. 2010). In diverse organisms, centrin (Ye et al. 2015), aurora kinase (Hu et al. 2014), a Ras protein (Castillo‐Lluva et al. 2010), and a trans‐Golgi transport protein (Mollapour et al. 2014) were identified as substrates for SUMO. These findings suggest that *Giardia* PV‐related proteins are substrates for GlSUMO and that the cargo system of *Giardia* is regulated through SUMOylation.

In conclusion, two Glcentrins were found to function in cell cycle progression and morphogenesis of *Giardia*. Our data demonstrate that both Glcents are substrates of GlSUMO, suggesting that SUMOylation is required for their proper localization. Our findings also showed that SUMOylation is involved in the regulation of vacuole formation in *Giardia*.

## Author Contributions

**Hye Rim Yeo:** investigation, validation, formal analysis, visualization. **Mee Young Shin:** data curation, investigation, validation. **Juri Kim:** conceptualization, methodology, data curation, investigation, formal analysis, supervision, visualization, writing – original draft, writing – review and editing. **Soon‐Jung Park:** conceptualization, methodology, funding acquisition, project administration, writing – original draft, writing – review and editing.

## Ethics Statement

The authors have nothing to report.

## Conflicts of Interest

None declared.

## Supporting information


**Figure S1:** Effect of the SUMOylation inhibitor, ginkgolic acid (GA), on the viability and cell cycle of *Giardia* trophozoites.

## Data Availability

The raw data that support the findings of this study are available from the lead author upon reasonable request.
