# Supplementary figures and images for: Two Centrins and Their Posttranslational Modification Modulate the Cell Cycle of Giardia lamblia
Source: Microbiologyopen. 2025 Jul 29;14(4):e70038. doi: 10.1002/mbo3.70038 (PMC12307541; doi:10.1002/mbo3.70038)

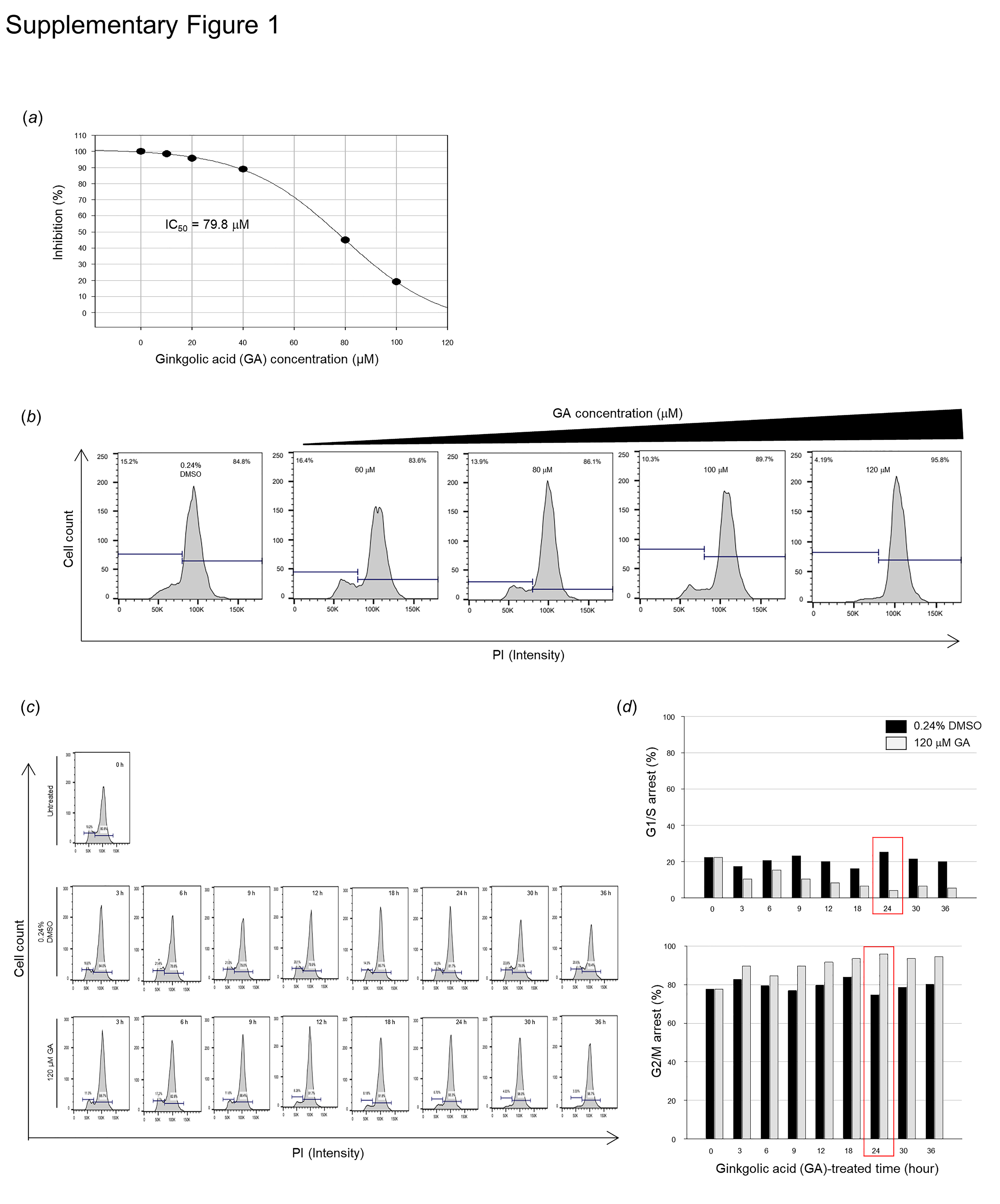

Supplement: Supplementary file 1 — Figure S1: Effect of the SUMOylation inhibitor, ginkgolic acid (GA), on the viability and cell cycle of Giardia trophozoites. [file MBO3-14-e70038-s001.tif]
